# NLRC3 negatively regulates CD4^+^ T cells and impacts protective immunity during *Mycobacterium tuberculosis* infection

**DOI:** 10.1371/journal.ppat.1007266

**Published:** 2018-08-22

**Authors:** Shengfeng Hu, Xialin Du, Yulan Huang, Yuling Fu, Yalong Yang, Xiaoxia Zhan, Wenting He, Qian Wen, Xinying Zhou, Chaoying Zhou, Xiao-Ping Zhong, Jiahui Yang, Wenjing Xiong, Ruining Wang, Yuchi Gao, Li Ma

**Affiliations:** 1 Institute of Molecular Immunology, School of Laboratory Medicine and Biotechnology, Southern Medical University, Guangzhou, China; 2 Department of laboratory medicine, The first Affiliated Hospital, Sun Yat-sen University, Guangzhou, China; 3 Department of Pediatrics, Division of Allergy and Immunology, Duke University Medical Center, Durham, NC, United States of America; Portland VA Medical Center, Oregon Health and Science University, UNITED STATES

## Abstract

NLRC3, a member of the NLR family, has been reported as a negative regulator of inflammatory signaling pathways in innate immune cells. However, the direct role of NLRC3 in modulation of CD4^+^ T-cell responses in infectious diseases has not been studied. In the present study, we showed that NLRC3 plays an intrinsic role by suppressing the CD4^+^ T cell phenotype in lung and spleen, including differentiation, activation, and proliferation. NLRC3 deficiency in CD4^+^ T cells enhanced the protective immune response against *Mycobacterium tuberculosis* infection. Finally, we demonstrated that NLRC3 deficiency promoted the activation, proliferation, and cytokine production of CD4^+^ T cells via negatively regulating the NF-κB and MEK-ERK signaling pathways. This study reveals a critical role of NLRC3 as a direct regulator of the adaptive immune response and its protective effects on immunity during *M*. *tuberculosis* infection. Our findings also suggested that NLRC3 serves as a potential target for therapeutic intervention against tuberculosis.

## Author summary

Accumulating evidence that NLRs play an intrinsic roles in regulating T cell responses in lungs and lymphoid tissues, however, to our known, contribution for NLRs modulation of T-cell responses directly in infectious diseases has not been studied. NLRC3, a member of the NLR family, has been reported as a negative regulator of inflammatory signaling pathways in innate immune cells. The direct role of NLRC3 in the modulation of CD4^+^ T-cell responses in infectious diseases has not been studied. In the present study, we focused on elucidating the roles of NLRC3 in shaping CD4^+^ T cell responses *in vivo* and *in vitro*, in particular during *M*. *tuberculosis* infection. We found that NLRC3 plays an intrinsic l roles by suppressing the CD4^+^ T cell phenotype in lungs and lymphoid tissues, including Th1 and Th17 differentiation, activation, and proliferation. NLRC3 deficiency in CD4^+^ T cells enhanced the protective immune response against *M*. *tuberculosis* infection. Finally, we demonstrated that NLRC3 deficiency promoted the activation, proliferation, and cytokine production of CD4^+^ T cells via negatively regulating the NF-κB and MEK-ERK signaling pathways. This study reveals a critical role of NLRC3 as a direct regulator of the adaptive immune response and its protective effects on immunity during *M*. *tuberculosis* infection. Our findings also suggested that NLRC3 serves as a potential target for therapeutic intervention against tuberculosis.

## Introduction

Nucleotide-binding oligomerization domain-like receptors (NLRs) belong to a large family of cytoplasmic sensors that act in response to host perturbation by infectious agents or cellular stress [1,2]. NLRs participate in a very diverse range of biological functions by regulating innate and acquired immune responses, and thus, contribute to immunity against infectious diseases. Some NLRs, such as NLRP3, NLRP7, and NLRC4, have been reported to promote the production of the proinflammatory cytokines, pro-IL-1β and pro-IL-18, via inflammasomes [3–5]. Other NLRs, such as NOD1, NOD2, NLRC5, and CIITA, are known to activate nuclear factor-κB (NF-κB), mitogen-activated protein kinases (MAPKs), and interferon (IFN) regulatory factors (IRFs) to stimulate innate immunity [6,7]. On the contrary, NLRP4, NLRP6, NLRP12, and NLRX1 have been demonstrated as negative regulators of inflammation [8–10]. In recent years, the roles of NLRs in regulating T cell responses have increasingly received research attention. Surprisingly, NLRP12 was found to act as a vital negative regulator of T-cell-mediated immunity and to influence NF-κB regulation and IL-4 production [11]. NOD1 and NOD2 promote the positive maturation of CD8 single-positive thymocytes in a thymocyte-intrinsic manner [12]. NLRP3 expression in CD4^+^ T cells was found to be required for completion of the inflammasome-mediated differentiation of T helper type 1 (Th1) and Th2 cells [13,14]. However, the exact roles of various NLR family members in regulating adaptive immune responses remain unclear.

NLRC3, an intracellular member of NLR family, has been reported to be expressed by various immune cell populations, including macrophages, epithelial cell and T cells and suppress inflammatory signaling pathways in innate immune cells [15–20]. These pathways include the NF-κB and STING pathways, which are exploited by bacteria or viruses to evade the host immune response and to promote survival [15,16]. A previous study showed that NLRC3 was significantly upregulated in T lymphocytes and suggested that NLRC3 suppresses T cell activation [21]. However, the exact role of NLRC3 and the mechanisms underlying its effects on CD4^+^ T cell activation and differentiation remain unclear, particularly during *in vivo* pathogen infection.

*Mycobacterium tuberculosis* (*M*. *tuberculosis*) is the primary causal pathogen of tuberculosis and has infected one-third of the world's population, making it a serious threat to human health. Various strategies, including improved diagnosis of active disease, drug therapy, and new vaccines, are required for effective management of tuberculosis infections [22,23]. Therefore, elucidating the immunoregulatory mechanisms underlying *M*. *tuberculosis* infection is an important step for the development of novel therapeutic approaches and for improving the success of vaccination strategies [24]. Previous studies have demonstrated that host resistance to *M*. *tuberculosis* infection requires the coordinated actions of the innate and adaptive immune cells [25]. Activation of bacilli-laden macrophages by cytokines secreted by effector CD4^+^ T cells, including IFN-γ, IL-17, TNF-α, and GM-CSF, is the key step for the immunoregulation of *M*. *tuberculosis* infection [26–28]. However, *M*. *tuberculosis* can evade immune responses by suppressing the activation of CD4^+^ T cells through factors, such as PD-1, CTLA4, and Tim-3 [29,30]. Pattern recognition receptors (PRRs) regulate innate and adaptive immune responses during *M*. *tuberculosis* infection via Toll-like receptors, C-type lectin receptors, and NLRs [31,32]. Therefore, there is a need to elucidate the mechanisms that lead to abnormalities in PRR signaling pathways that can influence disease pathogenesis during *M*. *tuberculosis* infection.

The present study focused on investigating the molecular mechanisms underlying NLRC3 regulation of CD4^+^ T-cell effector functions during *M*. *tuberculosis* infection. Our results revealed that negative regulation of CD4^+^ T cell activation by NLRC3 is mediated by the NF-κB and ERK signaling pathways both *in vitro* and *in vivo*. Furthermore, NLRC3 regulates negatively CD4^+^ T cell responses in lungs and lymphoid tissues, including differentiation and proliferation, which in turn suppresses the innate immune responses and promotes *M*. *tuberculosis* survival. NLRC3 was found to mediate immune evasion by *M*. *tuberculosis in vivo*. The above findings provided new insights showing that the suppression of NLRs inhibits the immune response to *M*. *tuberculosis* by controlling CD4^+^ T-cell immunity.

## Results

### NLRC3–deficient environment impacts CD4^+^ T cell phenotype during *M*. *tuberculosis* infection

**Fig 1:**
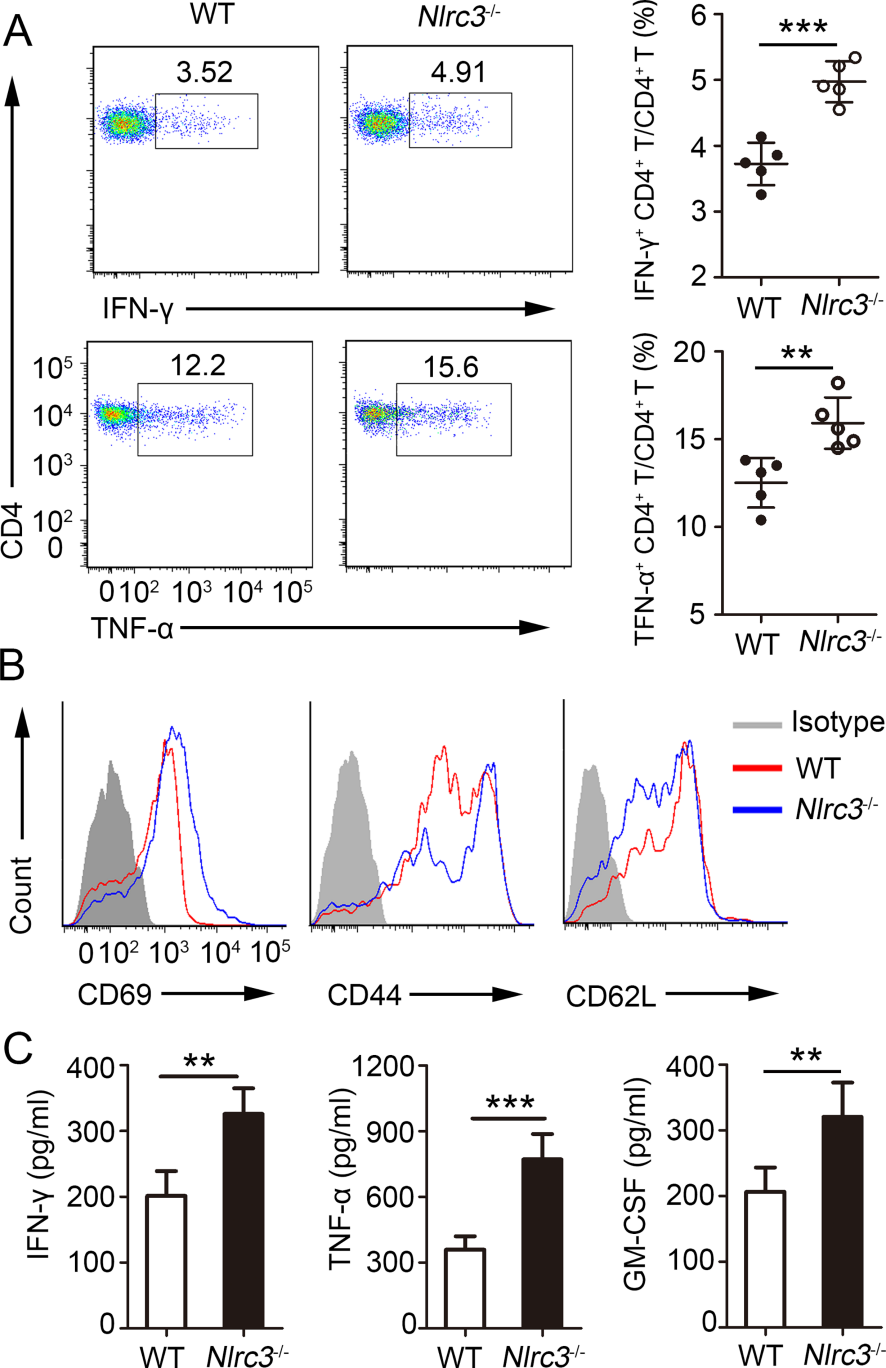
NLRC3 deficiency promotes activation of CD4^+^ T in *M*. *tuberculosis* infection. WT and *Nlrc3*^-/-^ mice were infected with *M*. *tuberculosis* and mice were harvested at 3 weeks post-infection (w.p.i.). **(A)** Lung cells were restimulated with *M*. *tuberculosis* lysate directly ex vivo and the intracellular production of IFN-γ and TNF-α by CD4+ T cells was determined. Pooled data are presented in the right panel. **(B)** Expression of activation markers by lung CD4^+^ T cells. **(C)** Concentration of IFN-γ, TNF-α and GM-CSF in lungs (homogenized in 2 ml PBS and 0.05% Tween 80) as detected by ELISA. Data shown in (A, C) are the mean ±SD. **P < 0.01 and ***P < 0.001. Data are representative of three independent experiments with similar results.

NLRC3 has emerged as a negative regulator of inflammatory signaling and is known to suppress the innate immune response during pathogen infection [16]. However, the ability of NLRC3 to modulate CD4^+^ T cell responses and the mechanisms by which NLRC3-mediated control of T cells can affect infectious disease progression remains poorly defined. Thus, to elucidate the effects of the NLRC3 knockdown on the CD4^+^ T cell phenotype during *M*. *tuberculosis* infection, we infected wild-type (WT) and NLRC3–deficient (*Nlrc3*^-/-^) mice with *M*. *tuberculosis*. CD4^+^ T cells isolated from the lungs of *Nlrc3*^-/-^ mice showed stronger expression of intracellular IFN-γ, TNF-α and IL-17A after restimulation than that showed by CD4^+^ T cells isolated from lungs of WT mice (Fig 1A, S1A Fig) at 3 weeks post-infection (w.p.i.). However, NLRC3 knockdown did not affect Treg cells (CD25^+^ Foxp3^+^) (S1B Fig). Activation analysis revealed that *Nlrc3*^-/-^ CD4^+^ T cells showed upregulation of CD44 and CD69 expression and downregulation of CD62L expression relative to that by WT CD4^+^ T cells (Fig 1B, S1C Fig). Consistent with these, the lungs of *Nlrc3*^-/-^ mice contained higher levels of IFN-γ and TNF-α than WT mice (Fig 1C). GM-CSF, a kind of main CD4^+^ T cell-derived cytokines that activates human and murine macrophages to inhibit intracellular *M*. *tuberculosis* growth [27], was also increased in lungs of *Nlrc3*^-/-^ mice (Fig 1C). Likewise, splenocytes isolated from *Nlrc3*^-/-^ mice with *M*. *tuberculosis* at 3 w.p.i. were also found to secrete increased amounts of IFN-γ and TNF-α after stimulation with ESAT-6 peptide (S1D Fig). Taken together, the above results demonstrate that NLRC3 is a negative regulator of CD4^+^ T cell activation during *M*. *tuberculosis* infection.

### *Nlrc3*^-/-^ mice are protected from *M*. *tuberculosis* infection

**Fig 2:**
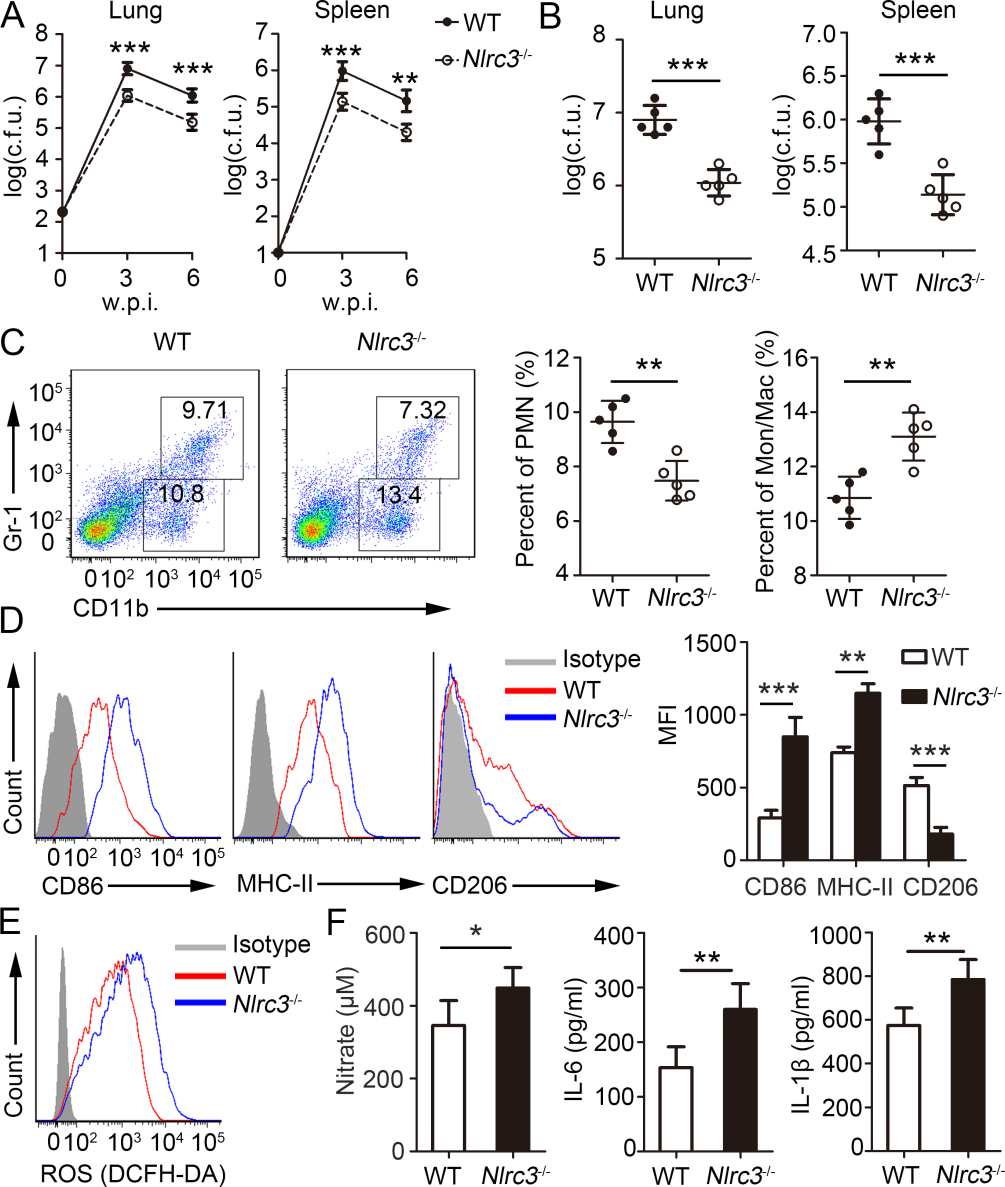
*Nlrc3*^-/-^ mice are protected from *M*. *tuberculosis* infection. WT and *Nlrc3*^-/-^ mice infected with approximately 200 colony-forming units (c.f.u.) of *M*. *tuberculosis* were monitored. **(A)** Bacterial burdens were determined after infection at 3 and 6 w.p.i.. **(B)** Bacterial burdens were determined after infection at 3 w.p.i.. **(C)** Frequencies of lung-infiltrating cells that are neutrophils (CD11b^+^ Gr-1^+^) or monocyte-macrophages (CD11b^+^ Gr-1^-^) at 3 w.p.i.. **(D)** Expressions of CD86, MHC-II and CD206 were detected on monocyte-macrophages (CD11b^+^ Gr-1^-^) via flow cytometry at 3 w.p.i.. **(E)** ROS production by monocyte-macrophages (CD11b^+^ Gr-1^-^) were detected assessed as mean fluorescence intensity (MFI) of intracellular CFDA. **(F)** Concentrations of nitrate were measured by nitrate reductase assay and concentrations of IL-6 and IL-1β in lungs (homogenized in 2 ml PBS and 0.05% Tween 80) were detected by ELISA at 3 w.p.i.. Data shown in are the mean ±SD. *P < 0.05, **P < 0.01 and ***P < 0.001. Data are representative of three independent experiments with similar results.

CD4^+^ T cells have been demonstrated to enhance the ability of macrophages to eliminate *M*. *tuberculosis* via the production of proinflammatory cytokines [25,33]. Given that CD4^+^ T cells showed enhanced activation phenotype under NLRC3-deficient conditions, we investigated the role of NLRC3 in the infectious disease. Previous studies showed that WT mice exposed to aerosol inoculation of *M*. *tuberculosis* showed improved control of bacterial burden and higher survival following CD4^+^ T recruitment into the lungs [34]. Consistent with the above findings, both WT and *Nlrc3*^-/-^ mice showed no significant weight loss (S2A Fig) and mortality (S2B Fig). However, bacterial titers in the lungs and spleens of *Nlrc3*^-/-^ mice were significantly lower than those of WT mice at 1, 3 and 6 w.p.i. (S2C Fig, Fig 2A and 2B). The bacterial burden in lungs from *Nlrc3*^-/-^ mice was reduced by about 80% relate to WT mice (Fig 2B). To assess the lung-infiltrating innate cells at 1 w.p.i., we found that proportions and numbers of monocyte-macrophages (CD11b^+^ Gr-1^-^) and polymorph nuclear neutrophils (PMN) (CD11b^+^ Gr-1^+^) had no difference between *Nlrc3*^-/-^ mice and WT mice (S2D and S2E Fig). The expression levels of surface marker CD86, MHC-II and CD206 on monocyte-macrophages were the similar between *Nlrc3*^-/-^ mice and WT mice at 1 w.p.i. (S2F Fig). The IL-6 levels in the lung homogenates of *Nlrc3*^-/-^ mice were significantly higher than those of WT mice, while IL-1β levels had no difference at 1 w.p.i. (S2G Fig). Moreover, we assessed the lung-infiltrating innate cells at 3 w.p.i., and found that total number of lung-infiltrating cells had no difference between *Nlrc3*^-/-^ mice and WT mice via histological observation and inflammatory cell count identified by flow cytometry (S3A And S3B Fig). However, *Nlrc3*^-/-^ mice contained significantly higher proportions and numbers of monocyte-macrophages, but lower proportions and numbers of PMN, relative to those in WT mice (Fig 2C, S3B Fig). Polarization of CD4^+^ T cells is known to influence macrophage differentiation *in vivo* [35]. We further assessed the effects of NLRC3 deficiency on macrophage differentiation. Surface marker expression analysis revealed that lung-infiltrating monocyte-macrophages in *Nlrc3*^-/-^ mice showed upregulation of CD86 and MHC-II expression and downregulation of CD206 expression relative to those of WT mice (Fig 2D), which suggested that macrophages tend to differentiate into classically activated macrophages under NLRC3-deficient conditions [36]. Consistent with the above results, macrophages collected from *Nlrc3*^-/-^ mice showed higher intracellular ROS levels than those collected from WT mice (Fig 2E). Nitrate, IL-6, and IL-1β levels in the lung homogenates of *Nlrc3*^-/-^ mice were significantly higher relative to those of WT mice (Fig 2F). Together, the above results indicate that NLRC3-deficient mice showed stronger immune responses.

### NRLC3 directly suppresses CD4^+^ T cell activation *in vitro*

To elucidate how NLRC3-deficient environment impacts CD4^+^ T cell phenotype, first we detected T cell development in *Nlrc3*^-/-^ mice. NLRC3 deficiency did not result in an obvious defect in thymic development (S4A and S4B Fig). However, the spleens had higher number of CD4^+^ T cells and CD8^+^ T cells (S4C and S4D Fig). Furthermore, CD4^+^ T cells from spleens of *Nlrc3*^-/-^ mice produced greater amounts of intracellular IFN-γ and IL-17 (S4E Fig). These results indicate that NLRC3 is dispensable for thymic development but suppresses the CD4^+^ T cell functions in spleens in naive mice.

**Fig 3:**
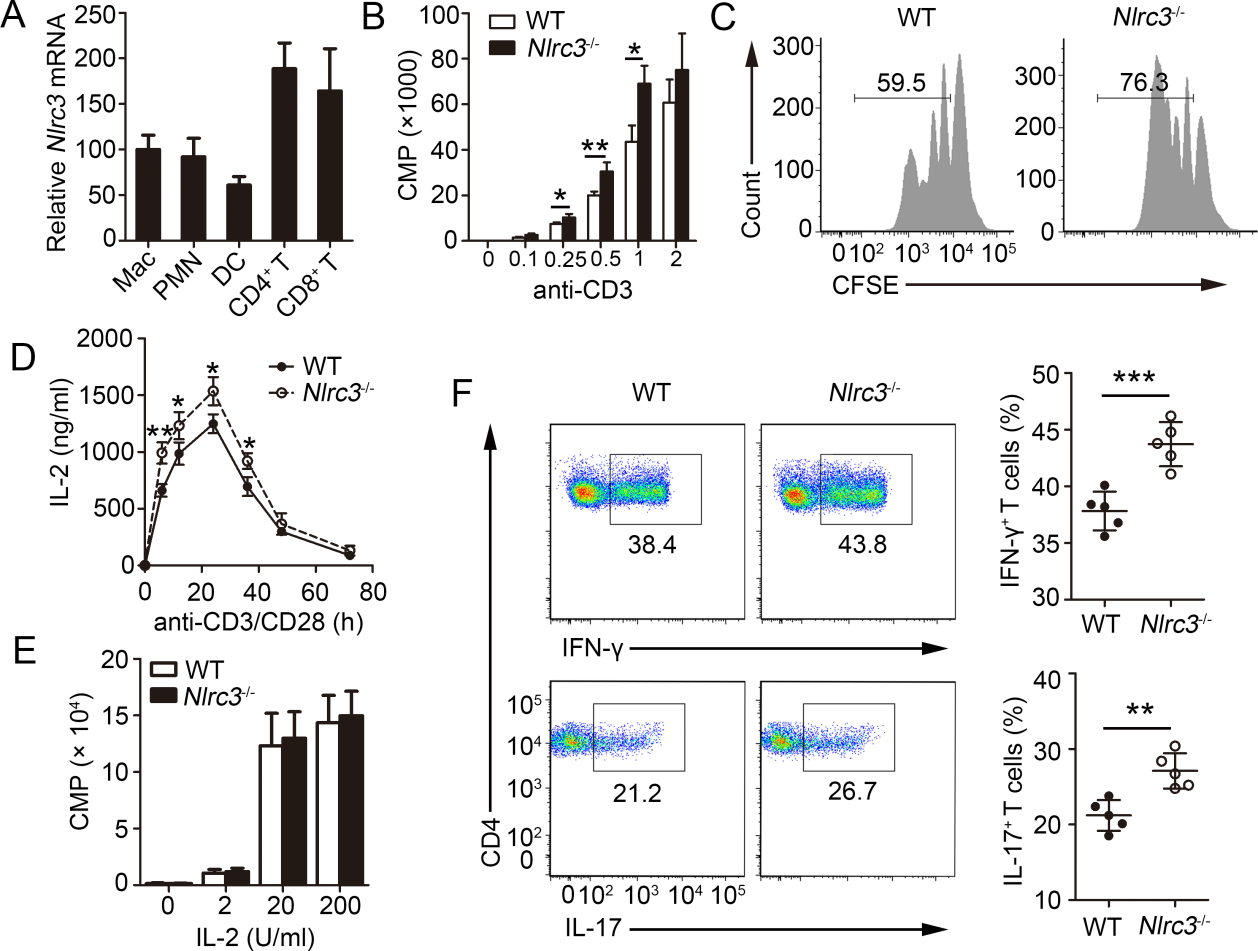
T cell activation *in vitro* requires NLRC3. **(A)** Relative expression of *Nlrc3* in purified macrophages (CD11b^+^ Gr-1^-^), dendritic cells (CD11c^+^ MHCII^hi^), polymorphonuclear leukocytes (PMNs) (CD11b^+^ Gr-1^+^), CD4^+^ T cells (CD3^+^ CD4^+^) and CD8^+^ T cells (CD3^+^ CD8^+^). **(B)** Purified naïve T cells isolated from WT and *Nlrc3*^-/-^ mice were stimulated with plate bound anti-CD3 (increasing concentrations) and anti-CD28 (1 μg/ml) for 48 hr and the incorporation of thymidine was measured during the final 8 hr. **(C)** Purified WT and *Nlrc3*^-/-^ naïve CD4^+^ T cells were labeled with CFSE and stimulated with anti-CD3 (1 μg/ml) and anti-CD28 (1 μg/ml) for 3 d. **(D)** Concentrations of IL-2 in supernatants of purified WT and *Nlrc3*^-/-^ naïve CD4^+^ T cells stimulated for 0–80 h with anti-CD3 (1 μg/ml) and anti-CD28 (1 μg/ml) were detected by ELISA. **(E)** Thymidine incorporation in purified WT and *Nlrc3*^-/-^ naive CD4^+^ T cells first primed with anti-CD3 and CD28 and then cultured with various concentrations of IL-2. **(F)** Purified WT and *Nlrc3*^-/-^ naïve CD4^+^ T cells were polarized in Th1 or Th17 culture conditions for 4 days. Data shown in (B, C, E, F) are the mean ±SD. *P < 0.05 and **P < 0.01. Data are representative of three independent experiments with similar results.

Next, we determined whether NLRC3 modulated CD4^+^ T development indirectly through antigen-presenting cells or directly. First, we evaluated expression of NLRC3 in various immune cell populations, and found that *Nlrc3* mRNA could be detected in various immune cell populations, especially the highest expression among T cells (Fig 3A). These results were consistent with previous report [21]. Thus, we hypothesized that is a direct regulator of CD4^+^ T cells. To confirm this hypothesis, naïve CD4^+^ T cells from WT and *Nlrc3*^-/-^ mice were activated *in vitro*. CD4^+^ T cell thymidine incorporation assays were conducted and found that purified *Nlrc3*^-/-^ CD4^+^ T cells displayed increased thymidine incorporation relative to WT CD4^+^ T cells (Fig 3B). Likewise, *Nlrc3*^-/-^ CD4^+^ T cells showed enhanced proliferation based on CFSE dye dilution (Fig 3C). IL-2 is a key cytokine that affects CD4^+^ T proliferation. Thus, we wondered whether the increased IL-2 production caused enhanced proliferation. To investigate this, we analyze the concentration of IL-2 in culture supernatant of CD4^+^ T with anti-CD3 and anti-CD28 antibodies. CD4^+^ T cells from *Nlrc3*^-/-^ mice produced more IL-2 than did WT CD4^+^ T cells (Fig 3D). Furthermore, we stimulated CD4^+^ T cells with anti-CD3 and anti-CD28, then washed the cells and recultured them with exogenous IL-2. The proliferation would be no markedly difference between WT and *Nlrc3*^-/-^ CD4^+^ T (Fig 3E). Our previous results showed that NLRC3-deficient environment impacts CD4^+^ T cell phenotype *in vivo*. We tested whether NLRC3-deficient in CD4^+^ T had the similar effect *in vitro*. The results showed that *Nlrc3*^-/-^ CD4^+^ T cells had higher intracellular levels of IFN-γ and IL-17, produced from *in vitro* polarized Th cells, than those in WT CD4^+^ T cells (Fig 3F). On the contrary, WT and *Nlrc3*^-/-^ CD4^+^ T cells showed no significant differences in intracellular IL-4 production (S5 Fig). Overall, these data indicate that NLRC3 suppresses activation and functions of CD4^+^ T directly.

### NRLC3 directly suppresses CD4^+^ T cell activation *in vivo*

**Fig 4:**
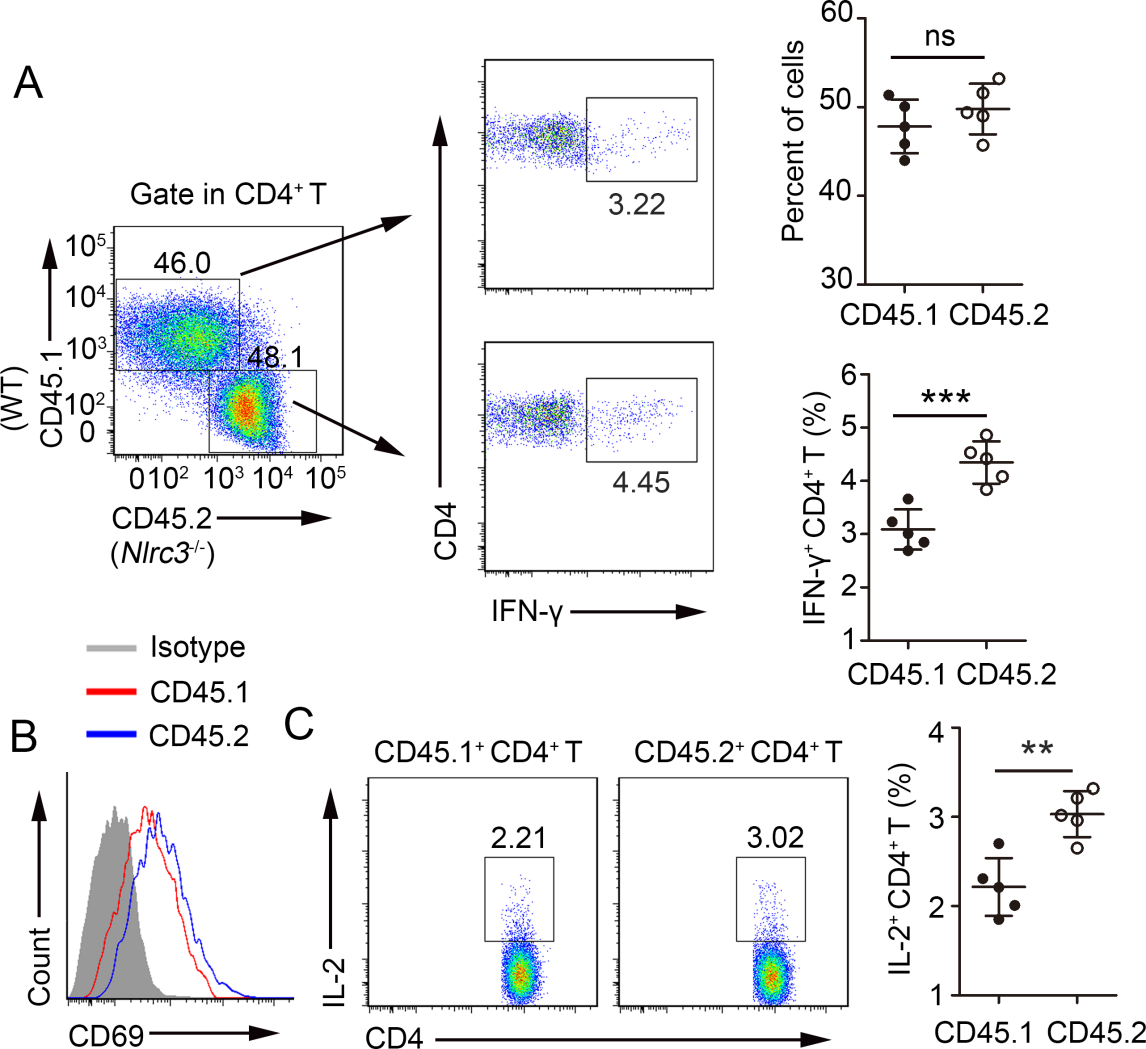
NLRC3 deficiency promotes differentiation of CD4^+^ T and IL-2 product*i*on *in vivo*. Naïve CD4^+^ T cells from CD45.1^+^ WT and CD45.2^+^ *Nlrc3*^-/-^ mice were mixed at a 1:1 ratio and competitively transferred via tail vein injection into *Rag2*^-/-^ recipient mice. Then recipient mice were infected with *M*. *tuberculosis* and were harvested at 3w.p.i.. **(A)** Lung cells were restimulated with *M*. *tuberculosis* lysate directly ex vivo and the intracellular production of IFN-γ by CD4+ T cells was determined. Representative FACs plots depicting gating of CD4^+^ T cells are shown. Gating strategies to evaluate cytokine production by WT and *Nlrc3*^-/-^ CD4^+^ T cells are provided. Numbers in the quadrants indicate the percent cells in each. Pooled data are presented in the right panel. **(B)** Expression of CD69 by lung CD4^+^ T cells. **(C)** Expression of IL-2 by lung CD4^+^ T cells. Pooled data are presented in the right panel. Data shown in (A, C) are the mean ±SD. *P < 0.05 and **P < 0.01. Data are representative of three independent experiments with similar results.

To further confirm the role of NLRC3 in the regulation of CD4^+^ T cells, competitive adoptive CD4^+^ T cell transfer assays were carried out. For these experiments, *Rag2*^-/-^ recipient mice received 1:1 CD45.1^+^ WT and CD45.2^+^ *Nlrc3*^-/-^ naïve CD4^+^ T cells and were subsequently infected with *M*. *tuberculosis*. We found that there was no significant difference in percentage of CD4^+^ T between CD45.1^+^ WT and CD45.2^+^ *Nlrc3*^-/-^ CD4^+^ T in the same environment (Fig 4A) at 3 w.p.i. However, the percentages of CD4^+^ T cells producing IFN-γ were higher in CD45.2^+^ *Nlrc3*^-/-^ CD4^+^ T cells than in CD45.1^+^ WT CD4^+^ T cells (Fig 4A). Furthermore, CD 69 expression was found to be upregulated in CD45.2^+^ *Nlrc3*^-/-^ CD4^+^ T cells (Fig 4B). Consistent with the *in vitro* results, *Nlrc3*^-/-^ CD4^+^ T cells showed higher IL-2 production after stimulation with *M*. *tuberculosis* lysate, thereby demonstrating the enhanced ability of CD45.2^+^ *Nlrc3*^-/-^ CD4^+^ T cells to produce IL-2 *in vivo* (Fig 4C). These results establish an intrinsic role for NLRC3 as a negative regulator of CD4^+^ T cell activation during *M*. *tuberculosis* infection.

### NLRC3 deficiency in CD4^+^ T cells promotes antibacterial immune responses

**Fig 5:**
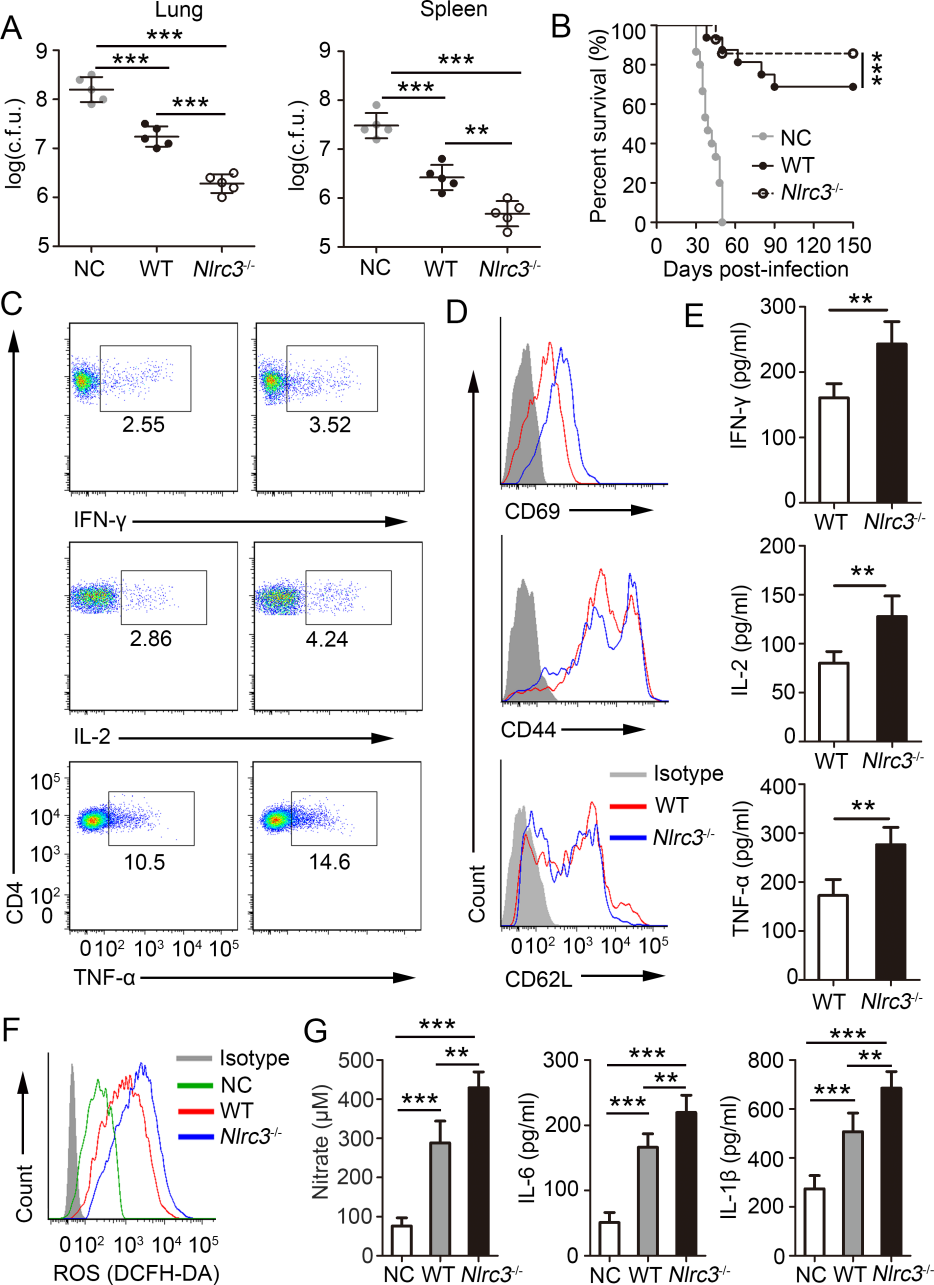
NLRC3 deficiency promotes the antibacterial ability *in vivo via* regulating CD4^+^ T cells. Purified WT or *Nlrc3*^-/-^ naïve CD4^+^ T cells were adoptively transferred into *Rag2*^*-/-*^ mice. Then recipient mice were infected with *M*. *tuberculosis* and parts of mice were harvested at 3w.p.i.. **(A)** Bacterial burdens were determined in lungs and spleen at 3w.p.i.. **(B)** Survival of mice every other day from 0–150 day post-infection (d.p.i.). **(C)** Lung cells were restimulated with *M*. *tuberculosis* lysate directly ex vivo and the intracellular production of IFN-γ, IL-2, and TNF-α by CD4^+^ T cells was determined. **(D)** Expression of activation markers by lung CD4^+^ T cells. **(E)** Concentration of IFN-γ, IL-2 and TNF-α in lungs (homogenized in 2 ml PBS and 0.05% Tween 80) as detected by ELISA. **(F)** ROS production by monocyte-macrophages (CD11b^+^ Gr-1^-^) were detected assessed as mean fluorescence intensity (MFI) of intracellular CFDA. **(G)** Concentrations of nitrate were measured by nitrate reductase assay and concentrations of IL-6 and IL-1β in lungs (homogenized in 2 ml PBS and 0.05% Tween 80) were detected by ELISA. Data shown in (A, E, G) are the mean ±SD. *P < 0.05, **P < 0.01 and ***P < 0.001. Data are representative of three independent experiments with similar results.

To define the role of NLRC3-deficiency of CD4^+^ T cells on *M*. *tuberculosis* infection, *Rag2*^-/-^ mice were injected with naïve CD4^+^ T cells from WT or *Nlrc3*^-/-^ mice and were subsequently infected with *M*. *tuberculosis*. We found that bacterial titres in the lungs and spleens of *Rag2*^-/-^ mice injected with the *Nlrc3*^-/-^ CD4^+^ T cells were significantly lower than those in mice injected with WT CD4^+^ T cells at 3 w.p.i. (Fig 5A). The survival of *Rag2*^-/-^ mice given *Nlrc3*^-/-^ CD4^+^ T cells was higher than that of *Rag2*^-/-^ mice given WT CD4^+^ T (Fig 5B). Furthermore, to detect activation and development of CD4^+^ T, we found that CD4^+^ T cells isolated from lungs of *Rag2*^-/-^ mice given *Nlrc3*^-/-^ CD4^+^ T expressed higher levels of intracellular IFN-γ, IL-2 and TNF-α after restimulation than those given WT CD4^+^T (Fig 5C, S6A Fig). CD4^+^ T cells isolated from the lungs of *Rag2*^-/-^ mice injected with *Nlrc3*^-/-^ CD4^+^ T cells showed stronger expression of CD69 and CD44, but weaker expression of CD62L, relative to those isolated from the lungs of *Rag2*^-/-^ mice injected with WT CD4^+^ T cells (Fig 5D, S6B Fig). Similarly, the lungs of *Rag2*^-/-^ mice injected with *Nlrc3*^-/-^ CD4^+^ T cells showed higher production of IFN-γ, IL-2 and TNF-α relative to those of WT mice (Fig 5E). In addition, CD4^+^ T cell counts in the draining lymph nodes (DLN), spleens, and lungs of *Rag2*^-/-^ mice injected with *Nlrc3*^-/-^ CD4^+^ T were higher than those of *Rag2*^-/-^ mice injected with WT CD4^+^ T cells (S6C Fig). We next evaluated lung-infiltrating innate cells, and results revealed that the lungs of *Rag2*^-/-^ mice injected with *Nlrc3*^-/-^ CD4^+^ T cells had significantly higher percentages of monocyte-macrophages, but lower percentages of PMNs, relative to those in the lungs of *Rag2*^-/-^ mice injected with WT CD4^+^ T cells (S7 Fig). Intracellular ROS in monocyte-macrophages (Fig 5F) and concentrations of nitrate, IL-6 and IL-1β in lung homogenate (Fig 5G) were all increased in *Rag2*^-/-^ mice given *Nlrc3*^-/-^ CD4^+^ T relative to those given WT CD4^+^ T. Together, these data indicate that absence of NLRC3 on CD4^+^ T cells promotes the antibacterial immune response of the body by regulating CD4^+^ T cell activation and further regulating the innate immune response.

### NLRC3 negatively regulates CD4^+^ T cell activation via NF-κB and ERK signaling

**Fig 6:**
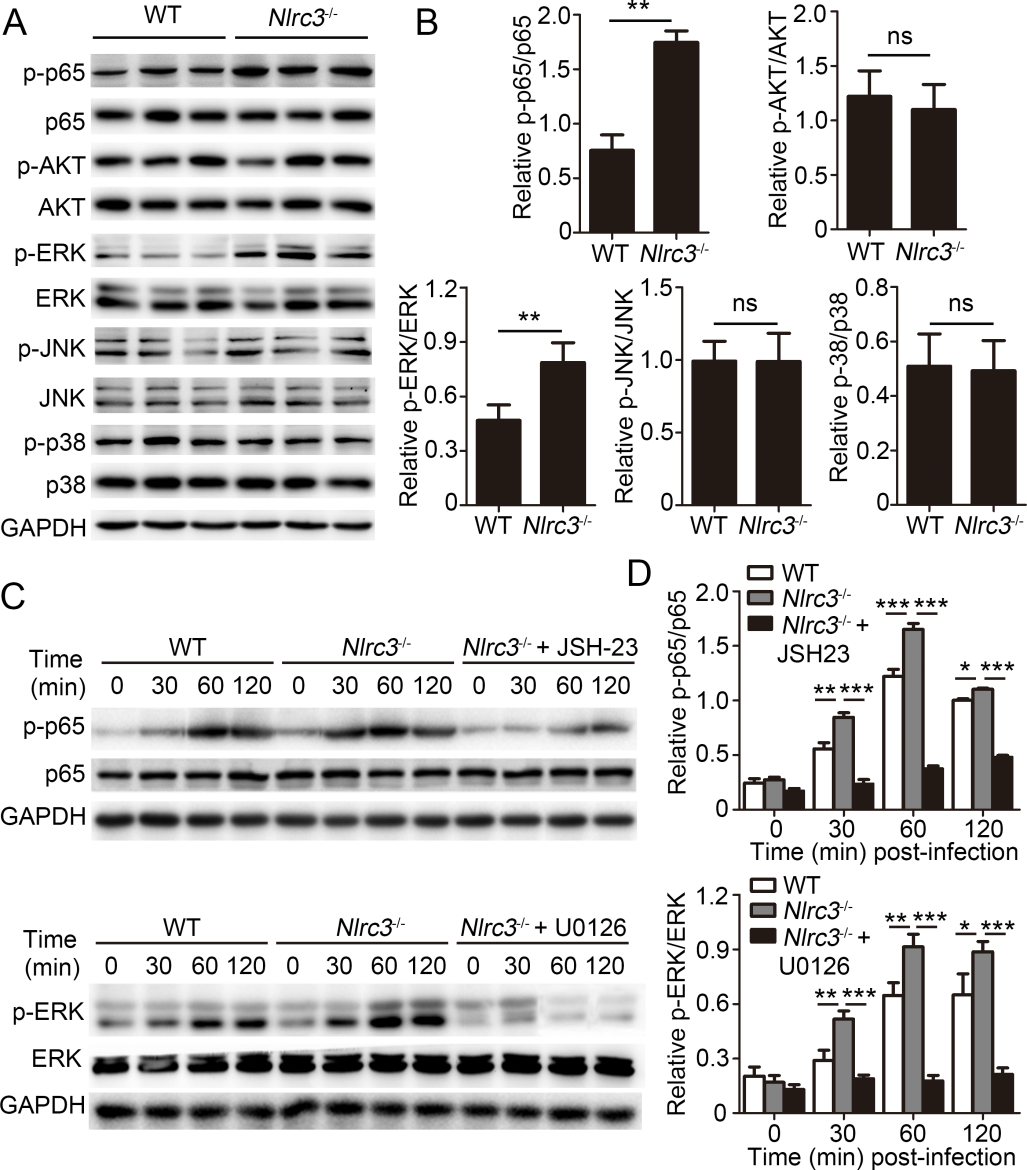
NLRC3 negatively regulates NF-κB and ERK Signaling in CD4^+^ T cells. **(A-B)** Purified WT or *Nlrc3*^-/-^ naïve CD4^+^ T cells were adoptively transferred into *Rag2*^*-/-*^ mice. Then recipient mice were infected with *M*. *tuberculosis* and were harvested at 3w.p.i.. Lungs were collected. **(A)** Immunoblot analysis of lung lysates. Each lane represents an individual mouse. **(B)** Densitometry quantification of band intensity for A. **(C-D)** Purified WT and *Nlrc3*^-/-^ CD4^+^ T cells were stimulated with anti-CD3 (1 μg/ml) plus anti-CD28 (1 μg/ml) in the presence or absence of the NF-κB inhibitor JSH-23 (20 μM) or MEK1/2-inhibitor U0126 (40 μM). **(C)** Lysates were probed for total and phosphorylated p65 (p-p65), p65, p-ERK, ERK and GAPDH. **(D)** Densitometry quantification of band intensity for C. Data shown in (B and D) are the mean ±SD. *P < 0.05, **P < 0.01 and ***P < 0.001. Data are representative of three independent experiments with similar results.

The specific molecular pathways that are controlled by NLRC3 in CD4^+^ T cells remain unclear. First, our results showed that transfer of *Nlrc3*^-/-^ CD4^+^ T cells resulted in enhanced phosphorylation of NF-κB p65 and ERK (Fig 6A and 6B) in lungs of recipient mice. While the phosphorylation of AKT, JNK and p38 was no difference between two groups (Fig 6A and 6B). These results suggested that increased phosphorylation of NF-κB p65 and ERK caused by NLRC3 deficiency of CD4^+^ T might contribute to improved antibacterial ability of the body. To determine whether NLRC3 suppressed activation of CD4^+^ T via mediating regulation of NF-κB p65 and ERK activation, we evaluated the signaling in purified CD4^+^ T cells. As expected, the results demonstrated that stimulation of *Nlrc3*^-/-^ CD4^+^ T cells with anti-CD3 and anti-CD28 led to increased phosphorylation of NF-κB p65 and ERK relative to WT CD4^+^ T cells (Fig 6C and 6D). Then we used NF-κB-inhibitor JSH-23 and MEK1/2-inhibitor U0126 to inhibit those pathway signals respectively, and observed phosphorylation of these proteins was decreased (Fig 6C and 6D).

**Fig 7:**
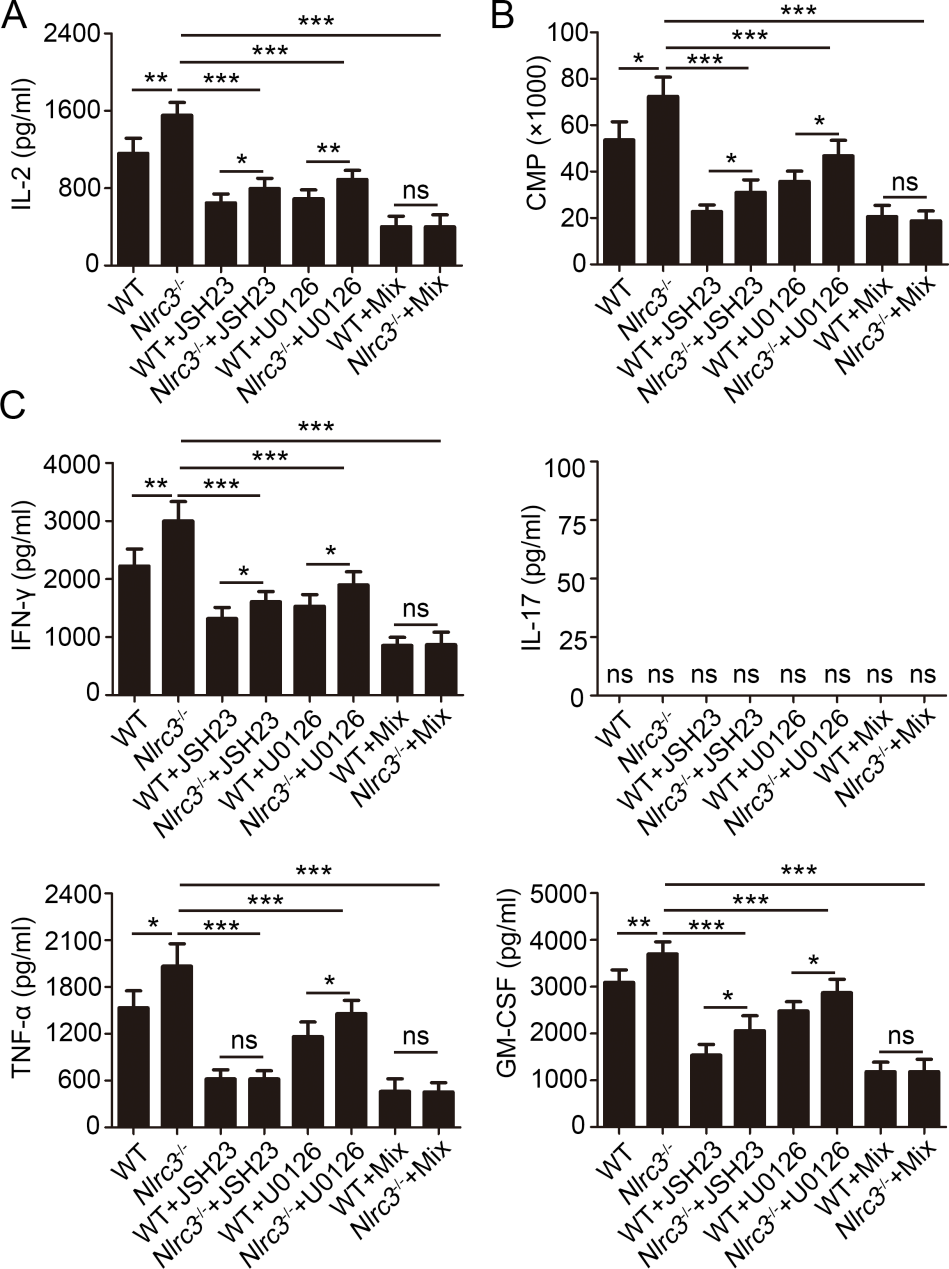
NLRC3 suppresses activation of CD4^+^ T cells via negatively regulating NF-κB and ERK Signaling. Purified WT and *Nlrc3*^-/-^ CD4^+^ T cells were stimulated for 48 hr with anti-CD3 (1 μg/ml) plus anti-CD28 (1 μg/ml) in the presence or absence of the NF-κB inhibitor JSH-23 (20 μM), MEK1/2-inhibitor U0126 (40 μM) or the mix of the two. **(A)** Concentrations of IL-2 in supernatants were detected by ELISA. **(B)** The incorporation of thymidine was measured during the final 8 hr. **(C)** Concentrations of IFN-γ, IL-17, TNF-α and GM-CSF in supernatants were detected by ELISA. Data shown are the mean ±SD. *P < 0.05, **P < 0.01 and ***P < 0.001. Data are representative of three independent experiments with similar results.

Next we examined the effect of inhibiting the phosphorylation of these proteins on activation of CD4^+^ T. Activation analysis revealed that *Nlrc3*^-/-^ CD4^+^ T cells showed upregulation CD69 expression after stimulated with anti-CD3 and anti-CD28 relative to that by WT CD4^+^ T cells, while CD69 expression level would have no difference between the *Nlrc3*^-/-^ CD4^+^ T and WT CD4^+^ T cells with mix of JSH-23 and U0126 (S8 Fig). Likewise, we found that mix of JSH-23 and U0126 suppressed increased IL-2 expression of *Nlrc3*^-/-^ CD4^+^ T cells stimulated with anti-CD3 and anti-CD28 (Fig 7A), but one of them alone could not limit completely increased IL-2 expression caused by NLRC3- deficiency on CD4^+^ T (Fig 7A). Likewise, pharmacological inhibition of NF-κB and ERK treatments lowered the enhanced proliferation capacity exhibited by *Nlrc3*^-/-^ CD4^+^ T (Fig 7B). Furthermore, pharmacological inhibitions of NF-κB and ERK were found to reduce IFN-γ, TNF-α and GM-CSF secretion by *Nlrc3*^-/-^ CD4^+^ T cells (Fig 7C). However, we did not detect the IL-17 secretion by CD4^+^ T cells stimulated only with anti-CD3 and anti-CD28 (Fig 7C). In total, NLRC3 negatively regulates activation of CD4^+^ T via mediating NF-κB and ERK signaling pathways.

## Discussion

Accumulating evidence that NLRs play an intrinsic role in regulating T cell responses lymphoid tissues [11,13]. However, to our known, contribution for NLRs modulation of T-cell responses directly in infectious diseases has not been studied. In the present study, we focused on elucidating the roles of NLRC3 in shaping CD4^+^ T cell responses *in vivo* and *in vitro*, in particular during *M*. *tuberculosis* infection. We found that NLRC3 played an intrinsic role in regulating negatively CD4^+^ T cell responses in lungs and lymphoid tissues, including differentiation and proliferation, which in turn suppresses the innate immune responses and promotes *M*. *tuberculosis* survival.

The current study provides a previously unidentified role that NLRC3 regulates Th1 and Th17 differentiation of CD4^+^ T during *M*. *tuberculosis* infection. NLRC3-deficient CD4^+^ T showed upregulated expression of Th1 and Th17-related cytokines, such as IFN-γ, IL-17, TNF-α and GM-CSF. These cytokines play vital role in activating macrophages to kill intracellular bacteria [24]. However, previous study showed that *M*. *tuberculosis* could induce immune evasion via T cell-costimulatory molecules, such as PD-1, CTLA4, and Tim-3 on T cells, to induce impairment of T-cell immunity [29,37,38]. The role of NLRC3 in inducing impairment of T-cell immunity is similar with T cell-costimulatory molecules. Thus, it might be an important strategy for *M*. *tuberculosis* to induce immune evasion via negative regulator of PRRs family suppressing adaptive immunity.

Following *M*. *tuberculosis* infection, phagocytic cells including macrophages and neutrophils will be recruited to the lung and form protective immune responses [39,40]. However, excessive PMN recruitment will cause damaging immunity and uncontrolled tissue damage [28]. NLRC3 has been described as a negative regulator of inflammatory signaling. Thus, it is worrying whether NLRC3-deficiency in body will lead to excessive inflammatory response. However, the results were almost completely opposite that PMN recruitment is reduced in NLRC3 deficient mice. Previous study showed that FN-γ directly inhibits neutrophil accumulation in lung and impairs neutrophil survival during *M*. *tuberculosis* infection [41]. Increased IFN-γ expression might contribute to the reduced PMN recruitment in NLRC3-deficient mice during *M*. *tuberculosis* infection.

Previous studies revealed that NLRC3 suppresses NF-κB signaling in macrophages and T cells [16,21]. We likewise observed that NLRC3-deficience in CD4^+^ T cells resulted in increased NF-κB p65 phosphorylation in CD4^+^ T cells. In addition, enhanced ERK phosphorylation was observed in NLRC3-deficient CD4^+^ T cells, which has not been reported in previous studies. Pharmacological inhibition of NF-κB or MEK-ERK signaling alone could not retune enhanced proliferation and cytokine production of CD4^+^ T cells induced by NLRC3-deficience. Thus, NLRC3 might regulate negatively activation of CD4^+^ T via suppressing both NF-κB and MEK-ERK signaling pathways. Previous study showed that NLRC3 inhibited activation of NF-κB by interacting with the adaptor TRAF6 to attenuate Lys63-linked ubiquitination of TRAF6 [15], but it is unclear whether NLRC3 regulates NF-κB signaling pathway via the same mechanism. The mechanism of NLRC3 regulating ERK signaling pathway remain to be investigated. Further studies are required to clarify these regulatory mechanisms. NLRC3 is a typical intracellular member of NLR family. However, the typical ligands of NLRC3 are still unclear. To study whether NLRC3 regulates NF-κB and ERK signal pathways via recognizing certain ligands will be very meaningful to enhance the understanding of its function.

In summary, we identified that NLRC3 regulated negatively CD4^+^ T cells directly. Loss of NLRC3 in CD4^+^ T cells enhanced the protective immune response against *M*. *tuberculosis* infection. Finally, we demonstrated that NLRC3-dificience in CD4^+^ T cells promoted the activation, proliferation and cytokine production of CD4^+^ T cell via negatively regulating NF-κB and MEK-ERK signaling pathways. Our study highlighted the critical role of NLRC3 in the regulation of the adaptive immune response and protective immunity during *M*. *tuberculosis* infection. Our findings also suggested that NLRC3 serves as a potential target for the development of therapeutic intervention against tuberculosis.

## Materials and methods

### Ethics statement

All animal experiments in this study were carried out in accordance with the recommendations in the Guide for the Care and Use of Laboratory Animals of the National Institutes of Health. All experimental protocols were reviewed and approved by the Medical Ethics Board and the Biosafety Management Committee of Southern Medical University (approval number SMU-L2015123).

### Mice

C57BL/6 mice were purchased from the Lab Animal Center of Southern Medicine University (Guangzhou, China). NLRC3-deficient (*Nlrc3*^-/-^) mice were built by Shanghai Research Center for Model Organisms (Shanghai, China). CD45.1^+^ and *Rag2*^*-/-*^ mice on a C57BL/6 background were purchased from Nanjing Biomedical Research Institute (Nanjing, China). All mice were maintained in the Lab Animal Center of Southern Medicine University under specific pathogen-free conditions.

### *M*. *tuberculosis* infection of mice

6~8-week old female and male mice were exposed to 1 × 10^7^ colony-forming units (CFU) of *M*. *tuberculosis* H37Rv (ATCC 27294, the same below) in an Inhalation Exposure System (Glas-Col, USA), which delivers ~ 200 bacteria to the lung per animal. At 24 h after infection, the bacterial titres in the lungs (left lobe) of at least two mice were determined to confirm the dose of *M*. *tuberculosis* H37Rv inoculation. Bacterial burden was determined by plating serial dilutions of lung (left lobe) and spleen homogenates onto 7H10 agar plates (BD Difco, USA) supplemented with 10% OADC. Plates were incubated at 37°C in 5% CO_2_ for 4 weeks before counting colonies.

### Lymphocyte isolation

A single-cell suspension was prepared from the spleen or lymph node by passing the organ through a 70-μm nylon cell strainer, followed by treatment with red blood cell lysis buffer. Lung cell suspensions (right lobe) were prepared by perfusing cold saline containing heparin through the heart, removed, and sectioned in ice-cold medium. Dissected lung tissue was incubated in 0.7 mg/ml collagenase IV and 30 μg/ml DNase (Sangon Biotech (Shanghai), China) at 37°C for 30 min. Digested lungs were disrupted by passage through a 70-μm nylon cell strainer, treated with red blood cell lysis buffer, and processed over a 40:80% Percoll (GE Healthcare) gradient. The resulting cell suspension was washed and counted.

### FACS analysis

For intracellular cytokine detection, isolated cells were cultured in 20 μg/ml of *M*. *tuberculosis* lysate or PMA/ionomycin for 1.5 h before 10 μg/ml Brefeldin A (eBioscience, USA) was added to the culture for 3.5 h more. For surface staining, lymphocytes were harvested, washed and stained for 30 min on ice with mixtures of fluorescently conjugated mAbs or isotype-matched controls. mAbs of mice were as follows: APC-Cy7-anti-CD3, PE-Cy7-anti-CD4, APC-anti-CD8a, PE-anti-CD45.1, Alexa Fluor 700-anti-CD45.2, PE-anti-CD25, PerCP-cy5.5-anti-CD69, PE-anti-CD44, FITC-anti-CD62L, PE-cy7-anti-Gr-1, PE-anti-CD11b, APC-ant-CD86, Alexa Fluor 700-anti-MHC-II and PerCP-cy5.5-anti-CD206 (eBioscience). For intracellular staining, the cells were incubated 20 min in IC Fixation buffer (eBioscience), followed by permeabilization buffer (eBioscience) and 1 h of incubation with appropriate mAbs of mice: PE-anti-IFN-γ, FITC-anti-IL-17, PerCP-cy5.5-anti-IL-4, FITC-anti-IL-2 and FITC-anti-Foxp3. Cell phenotype was analyzed by flow cytometry on a flow cytometer (BD LSR II) (BD Biosciences, USA). Data were acquired as the fraction of labeled cells within a live-cell gate and analyzed using FlowJo software (Tree Star). All gates were set on the basis of isotype-matched control antibodies.

### Enzyme-linked immunosorbent assay (ELISA)

Lungs (right lobe) were homogenized in 2 ml PBS + 0.05% Tween 80. Homogenized tissue supernatants were filtered (0.22 μ m). Cell culture supernatants were collected. Cytokine production was measured by enzyme-linked immunosorbent assay of mouse IFN-γ, TNF-α, GM-CSF, nitrate, IL-6, IL-1β or IL-2 (ExCell Bio, China) according to the manufacturer’s protocol.

### *Nlrc3* expression

Macrophages (Gr-1^-^ CD11b^+^), polymorphonuclear leukocytes (PMNs) (Gr-1^+^ CD11b^+^), dendritic cells (CD11c^+^ MHC-II^hi^), CD4^+^ T cells (CD3^+^ CD4^+^) and CD8^+^ T cells (CD3^+^ CD8^+^) were purified by flow cytometry sorting and total RNA was isolated with Trizol (Invitrogen, USA) according to the manufacturer’s instructions. 1 mg of RNA was reverse transcribed to cDNA with random RNAspecific primers using the high-capacity cDNA reverse transcription kit (Applied Biosystems). Transcript amounts of *Nlrc3* and *Gapdh* were analyzed with SYBR-Green (Applied Biosystems) according to the manufacturer’s recommendations. The primer sequences used for PCR are in S1 Table.

### Thymidine incorporation assay

Splenocytes and LNs were harvested from WT and *Nlrc3*^-/-^mice. Naïve WT and *Nlrc3*^-/-^ CD4^+^ T cells (CD3^+^ CD4^+^ CD44^lo^ CD62L^hi^) were purified by FACs sorting. Purified naive T cells were stimulated with increasing concentrations of plate bound anti-CD3 (eBioscience) and anti-CD28 (eBioscience) in triplicate wells for 48 hr. During the last 8 hr of stimulation, T cells were pulsed with [^3^H]thymidine and the amount of incorporated [^3^H]thymidine was measured as counts per minute (cpm). For signal pathway-inhibition studies, the NF-κB-inhibitor JSH-23 (20μM) and MEK1/2-inhibitor U0126 (40μM) (Selleck, USA) was added into culture media.

### CFSE CD4^+^ T cell proliferation assay

Splenocytes and LNs were harvested from WT and *Nlrc3*^-/-^mice. Naïve WT and *Nlrc3*^-/-^ CD4^+^ T cells (CD3^+^ CD4^+^ CD44^lo^ CD62L^hi^) were purified by FACs sorting. Purified T cells were labeled with 2.5 μM CFSE and then 5 × 10^4^ T cells/well were stimulated with anti-CD3 (1.0 μg/ml) and anti-CD28 (1.0 μg/ml). T cells were cultured for 72 hrs and proliferation was determined by Flow cytometry analysis of CFSE dilution.

### Th cells polarization

For Th cell polarization, splenocytes and LNs were harvested from WT and *Nlrc3*^-/-^mice. Naïve WT and *Nlrc3*^-/-^ CD4^+^ T cells (CD3^+^ CD4^+^ CD44^lo^ CD62L^hi^) were purified by FACs sorting. Cells were cultured in RPMI 1640 medium (Thermo Fisher Scientific, USA) with plate-bound anti-CD3 and anti-CD28 antibodies in the presence of cytokines (R&D, USA) and neutralizing antibodies (R&D) as follows. Th1 conditions: IFN-γ (200 ng/ml), IL-12 (2 ng/ml), anti-IL-4 (5 mg/ml). Th2 conditions: IL-4 (10 ng/ml) and anti-IFN-γ (5 mg/ml). Th17 conditions: TGF-β3 (2ng/ml), IL-6 (25 ng/ml), IL-1β (10 ng/ml), IL-23 (10 ng/ml), anti-IL-4 (5 mg/ml) and anti-IFN-γ (5 mg/ml).

### CD4^+^ T cells adoptive transfer

Splenocytes and LNs were harvested from WT and *Nlrc3*^-/-^mice. Naïve WT and *Nlrc3*^-/-^ CD4^+^ T cells (CD3^+^ CD4^+^ CD44^lo^ CD62L^hi^) were purified by FACs sorting. Cells were counted and then (1x10^6^ cells) adoptively transferred into *Rag2*^-/-^ recipient mice via tail vein injection. These recipient mice were infected with *M*. *tuberculosis* after one day.

### Competitive T cell adoptive transfer

Splenocytes and LNs were harvested from WT (CD45.1^+^) and *Nlrc3*^-/-^ (CD45.2^+^) mice. Naïve WT and *Nlrc3*^-/-^ CD4^+^ T cells (CD3^+^ CD4^+^ CD44^lo^ CD62L^hi^) were purified by FACs sorting and mixed at a 1:1 ratio. Mixed 1:1 naïve T cells (1x10^6^ total cells) were then adoptively transferred into *Rag2*^-/-^ recipient mice via tail vein injection. These recipient mice were infected with *M*. *tuberculosis* after one day. Lungs were harvested on day 21 post adoptive transfer and homeostatic expansion was evaluated using congenic CD45 markers and flow cytometry.

### Western blotting

Tissue or cells were washed three times with ice-cold PBS and then lysed in lysis buffer containing 1 mM phenylmethylsulfonyl fluoride, 1% (vol/vol) protease inhibitor cocktail (Sigma Aldrich, USA), and 1 mM DTT. Equal amounts (20 mg) of cell lysates were resolved using 8–15% polyacrylamide gels transferred to PVDF membrane. Membranes were blocked in 5% non-fat dry milk in PBST and incubated overnight with the respective primary antibodies at 4°C. The membranes were incubated at room temperature for 1 h with appropriate HRP-conjugated secondary antibodies and visualized with Plus-ECL (PerkinElmer, CA) according to the manufacturer’s protocol.

### Statistics

All experiments were performed at least twice. When shown, multiple samples represent biological (not technical) replicates of mice randomly sorted into each experimental group. No blinding was performed during animal experiments. Determination of statistical differences was performed with Prism 5 (Graphpad Software, Inc.) using unpaired two-tailed *t*-tests (to compare two groups with similar variances), or one-way ANOVA with Bonferonni’s multiple comparison test (to compare more than two groups).

## Supporting information

S1 Fig Related to Fig 1. **NLRC3 deficiency promotes activation of CD4**^**+**^ **T in *M*. *tuberculosis* infection.** WT and *Nlrc3*^-/-^ mice were infected with *M*. *tuberculosis* and mice were harvested at 3 weeks post-infection (w.p.i.). **(A)** Lung cells were restimulated with *M*. *tuberculosis* lysate directly ex vivo and the intracellular production of IL-17 by CD4+ T cells was determined. Pooled data are presented in the right panel. **(B)** Expression of CD25 and Foxp3 were detected on CD4^+^ T cells from lungs. Pooled data are presented in the right panel. **(C)** Expression of activation markers were assessed as mean fluorescence intensity (MFI) on lung CD4^+^ T cells. **(D)** Splenocytes were stimulated for 48 hr with ESAT-6 peptide (5μg/ml) and cytokine production was measured by ELISA. Data shown are the mean ±SD. **P < 0.01 and ***P < 0.001. Data are representative of three independent experiments with similar results.
(TIF)

S2 Fig Related to Fig 2. **NLRC3 deficiency had no effect on weight and survival of mice wi**th *M*. *tuberculosis* infe**ction.** Mice infected with approximately 200 colony-forming units of *M*. *tuberculosis* were monitored at various days post infection. **(A)** Weight change. **(B)** Survival every day from 0–200 day post-infection. Data shown are the mean ±SD. **(C)** Bacterial burdens were determined after infection at 1w.p.i.. **(D)** Frequencies of lung-infiltrating cells that are neutrophils (CD11b^+^ Gr-1^+^) or monocyte-macrophages (CD11b^+^ Gr-1^-^) at 1 w.p.i.. **(E)** Numbers of lung-infiltrating cells were counted at 1 w.p.i. **(F)** Expressions of CD86, MHC-II and CD206 were detected on monocyte-macrophages (CD11b^+^ Gr-1^-^) via flow cytometry at 1 w.p.i.. **(G)** Concentrations of IL-6 and IL-1β in lungs (homogenized in 2 ml PBS and 0.05% Tween 80) were detected by ELISA at 1 w.p.i.. Data shown are the mean ±SD. **P < 0.01. Data are representative of three independent experiments with similar results.
(TIF)

S3 Fig Related to Fig 2. WT and *Nlrc3*^-/-^ mice infected with approximately 200 colony-forming units of *M*. *tuberculosis* were monitored. **(A)** H&E-stained lung sections derived from two representative mice in each group of mice 3 w.p.i.. The magnification is shown at the right of each image. **(B)** Numbers of lung-infiltrating cells were counted at. Data shown are the mean ±SD. *P < 0.05 and **P < 0.01. Data are representative of three independent experiments with similar results.
(TIF)

S4 Fig Related to Fig 3. **NLRC3 does not affect thymic development but does influence mature CD4**^**+**^ **T cells. (A)** Representative expression of CD4 and CD8 by WT and *Nlrc3*^-/-^ thymocytes. Pooled data are presented in the right panel. DN, double negative (CD4^-^ CD8^-^); DP, double positive (CD4^+^ CD8^+^); CD4, CD4 single positive (CD4^+^ CD8^-^); CD8, CD8 single positive (CD4^-^ CD8^+^). **(B)** Total numbers of thymocytes in each stage of thymic development. **(C)** Representative expression of splenic CD4^+^ and CD8^+^ T cells from WT and *Nlrc3*^-/-^ mice. Pooled data are presented in the right panel. **(D)** Enumeration of splenic CD4^+^ and CD8^+^ T cells from WT and *Nlrc3*^-/-^ mice. **(E)** Production of IFN-γ and IL-17 by CD4+ T cells following stimulation with PMA/ionomycin. Pooled data are presented in the right panel. Data shown are the mean ±SD. *P < 0.05 and **P < 0.01. Data are representative of three independent experiments with similar results.
(TIF)

S5 Fig Related to Fig 3. **NLRC3 does not affect differentiation of Th2.** Purified WT and *Nlrc3*^-/-^ naïve CD4^+^ T cells were polarized in Th2 culture conditions for 4 days. Data shown are the mean ±SD. Data are representative of three independent experiments with similar results.
(TIF)

S6 Fig Related to Fig 5. **NLRC3 deficiency of CD4**^**+**^ **T promotes the antibacterial ability *in vivo*.** Purified WT or *Nlrc3*^-/-^ naïve CD4^+^ T cells were adoptively transferred into *Rag2*^*-/-*^ mice. Then recipient mice were infected with *M*. *tuberculosis* and parts of mice were harvested at 3w.p.i.. **(A)** Lung cells were restimulated with *M*. *tuberculosis* lysate directly *ex vivo* and the intracellular production of IFN-γ, IL-2, and TNF-α by CD4^+^ T cells was determined. Pooled data are presented. **(B)** Mean fluorescence intensity (MFI) of activation markers by lung CD4^+^ T cells. **(C)** Enumeration of CD4^+^ cells in draining lymph nodes (DLNs), spleens and lungs. Data shown are the mean ±SD. **P < 0.01 and ***P < 0.001. Data are representative of three independent experiments with similar results.
(TIF)

S7 Fig Related to Fig 5. **NLRC3 deficiency of CD4**^**+**^ **T affected infiltration of myeloid cells to lung.** Purified WT or *Nlrc3*^-/-^ naïve CD4^+^ T cells were adoptively transferred into *Rag2*^*-/-*^ mice. Then recipient mice were infected with *M*. *tuberculosis* and parts of mice were harvested at 3w.p.i.. Frequencies of lung-infiltrating cells that are neutrophils (CD11b^+^ Gr-1^+^) or monocyte-macrophages (CD11b^+^ Gr-1^-^). Pooled data are presented in the right panel. Data shown are the mean ±SD. **P < 0.01. Data are representative of three independent experiments with similar results.
(TIF)

S8 Fig Related to Fig 7. **NLRC3 suppresses activation of CD4**^**+**^ **T cells via negatively regulating NF-κB and ERK Signaling.** Purified WT and *Nlrc3*^-/-^ CD4^+^ T cells were stimulated for 48 hr with anti-CD3 (1 μg/ml) plus anti-CD28 (1 μg/ml) in the presence or absence of the NF-κB inhibitor JSH-23 (20 μM), MEK1/2-inhibitor U0126 (40 μM) or the mix of the two. And then expression of CD69 were detected. *P < 0.05 and **P < 0.01. Data are representative of three independent experiments with similar results.
(TIF)

S1 Table The primers of RT-PCR. (XLSX)
