# Supplementary figures and images for: NLRC3 negatively regulates CD4+ T cells and impacts protective immunity during Mycobacterium tuberculosis infection
Source: PLoS Pathog. 2018 Aug 22;14(8):e1007266. doi: 10.1371/journal.ppat.1007266 (PMC6122840; doi:10.1371/journal.ppat.1007266)

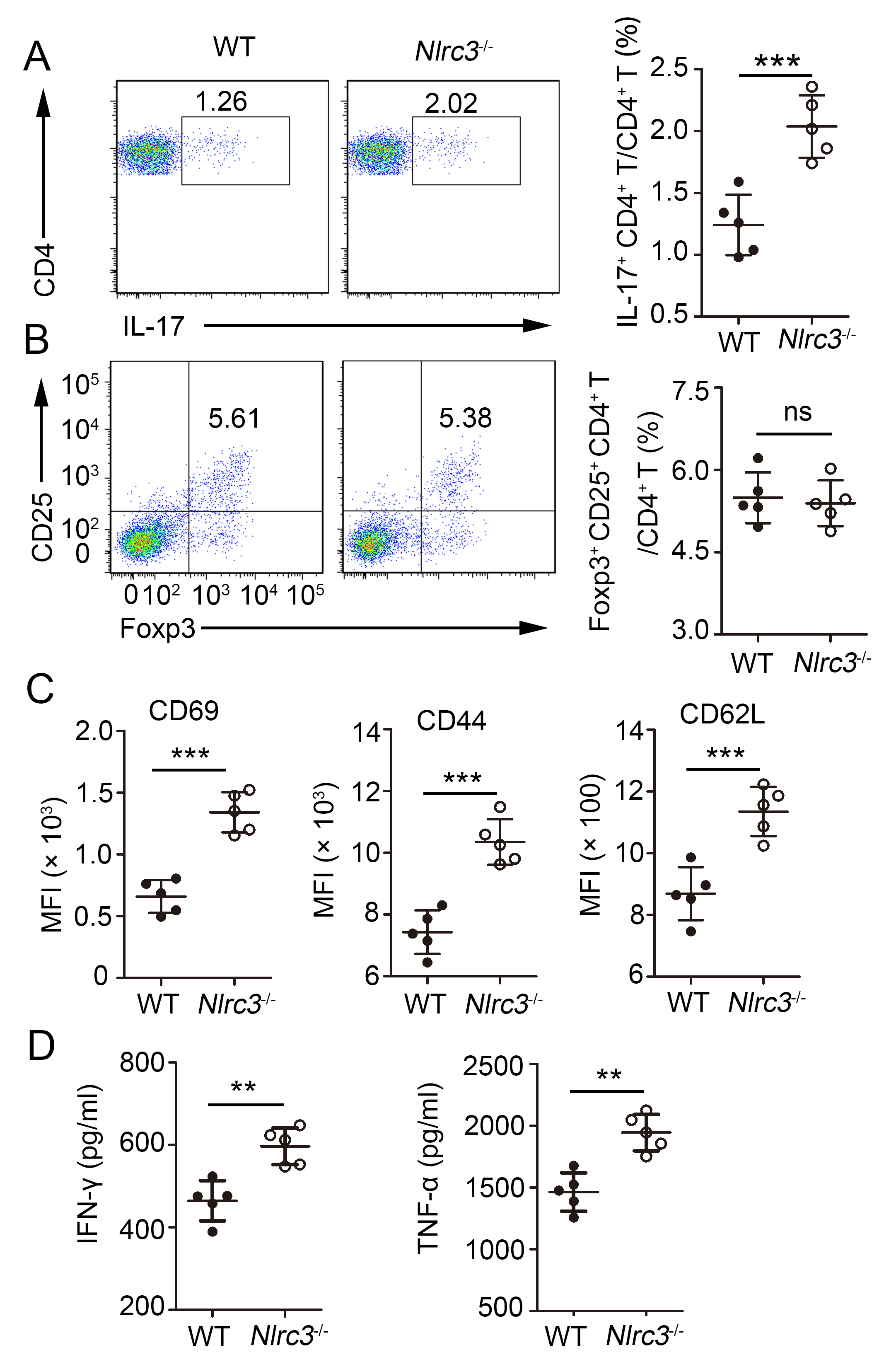

Supplement: S1 Fig — NLRC3 deficiency promotes activation of CD4+ T in M. tuberculosis infection. WT and Nlrc3-/- mice were infected with M. tuberculosis and mice were harvested at 3 weeks post-infection (w.p.i.). (A) Lung cells were restimulated with M. tuberculosis lysate directly ex vivo and the intracellular production of IL-17 by CD4+ T cells was determined. Pooled data are presented in the right panel. (B) Expression of CD25 and Foxp3 were detected on CD4+ T cells from lungs. Pooled data are presented in the right panel. (C) Expression of activation markers were assessed as mean fluorescence intensity (MFI) on lung CD4+ T cells. (D) Splenocytes were stimulated for 48 hr with ESAT-6 peptide (5μg/ml) and cytokine production was measured by ELISA. Data shown are the mean ±SD. **P < 0.01 and ***P < 0.001. Data are representative of three independent experiments with similar results. (TIF) [file ppat.1007266.s001.tif]

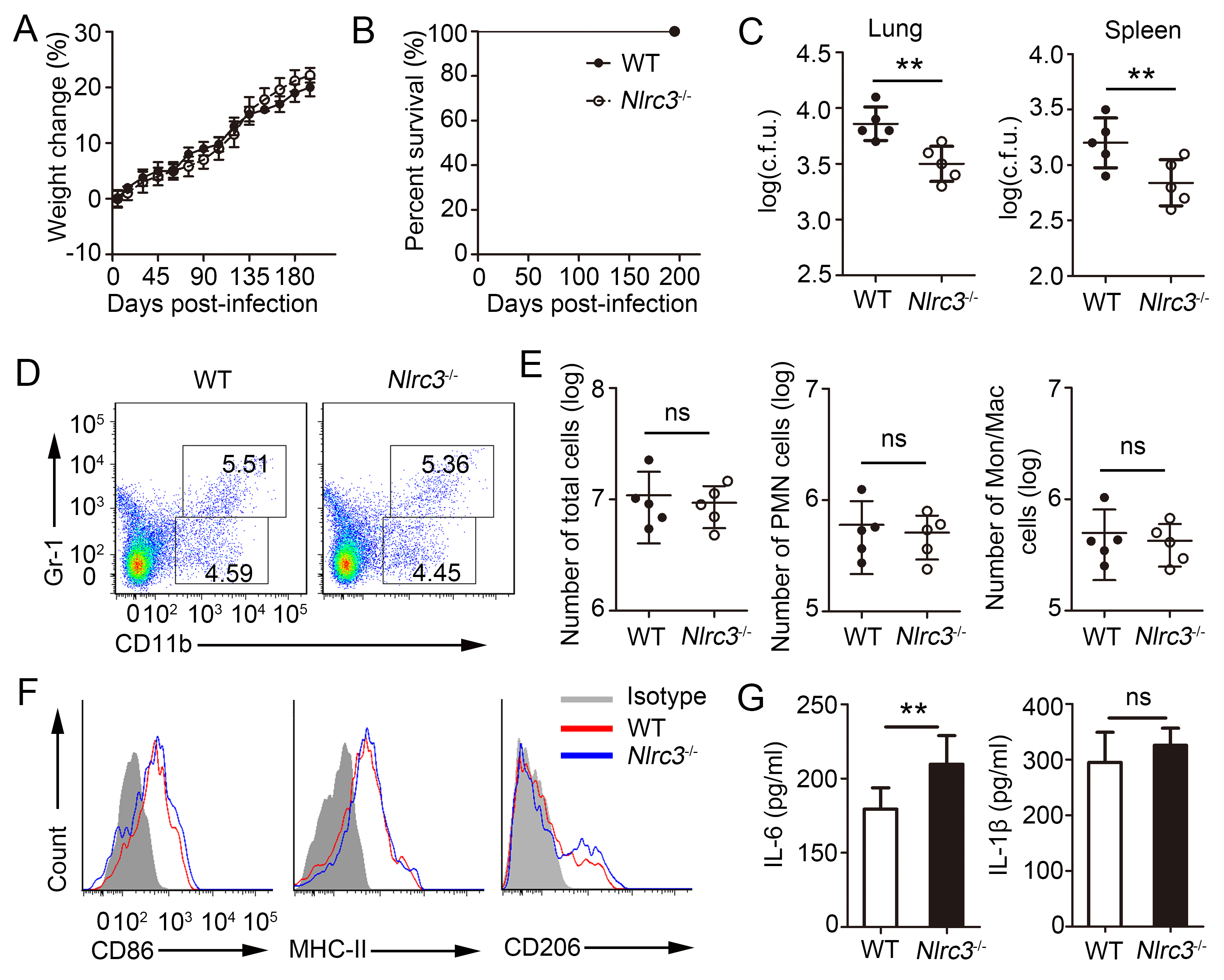

Supplement: S2 Fig — NLRC3 deficiency had no effect on weight and survival of mice with M. tuberculosis infection. Mice infected with approximately 200 colony-forming units of M. tuberculosis were monitored at various days post infection. (A) Weight change. (B) Survival every day from 0–200 day post-infection. Data shown are the mean ±SD. (C) Bacterial burdens were determined after infection at 1w.p.i.. (D) Frequencies of lung-infiltrating cells that are neutrophils (CD11b+ Gr-1+) or monocyte-macrophages (CD11b+ Gr-1-) at 1 w.p.i.. (E) Numbers of lung-infiltrating cells were counted at 1 w.p.i. (F) Expressions of CD86, MHC-II and CD206 were detected on monocyte-macrophages (CD11b+ Gr-1-) via flow cytometry at 1 w.p.i.. (G) Concentrations of IL-6 and IL-1β in lungs (homogenized in 2 ml PBS and 0.05% Tween 80) were detected by ELISA at 1 w.p.i.. Data shown are the mean ±SD. **P < 0.01. Data are representative of three independent experiments with similar results. (TIF) [file ppat.1007266.s002.tif]

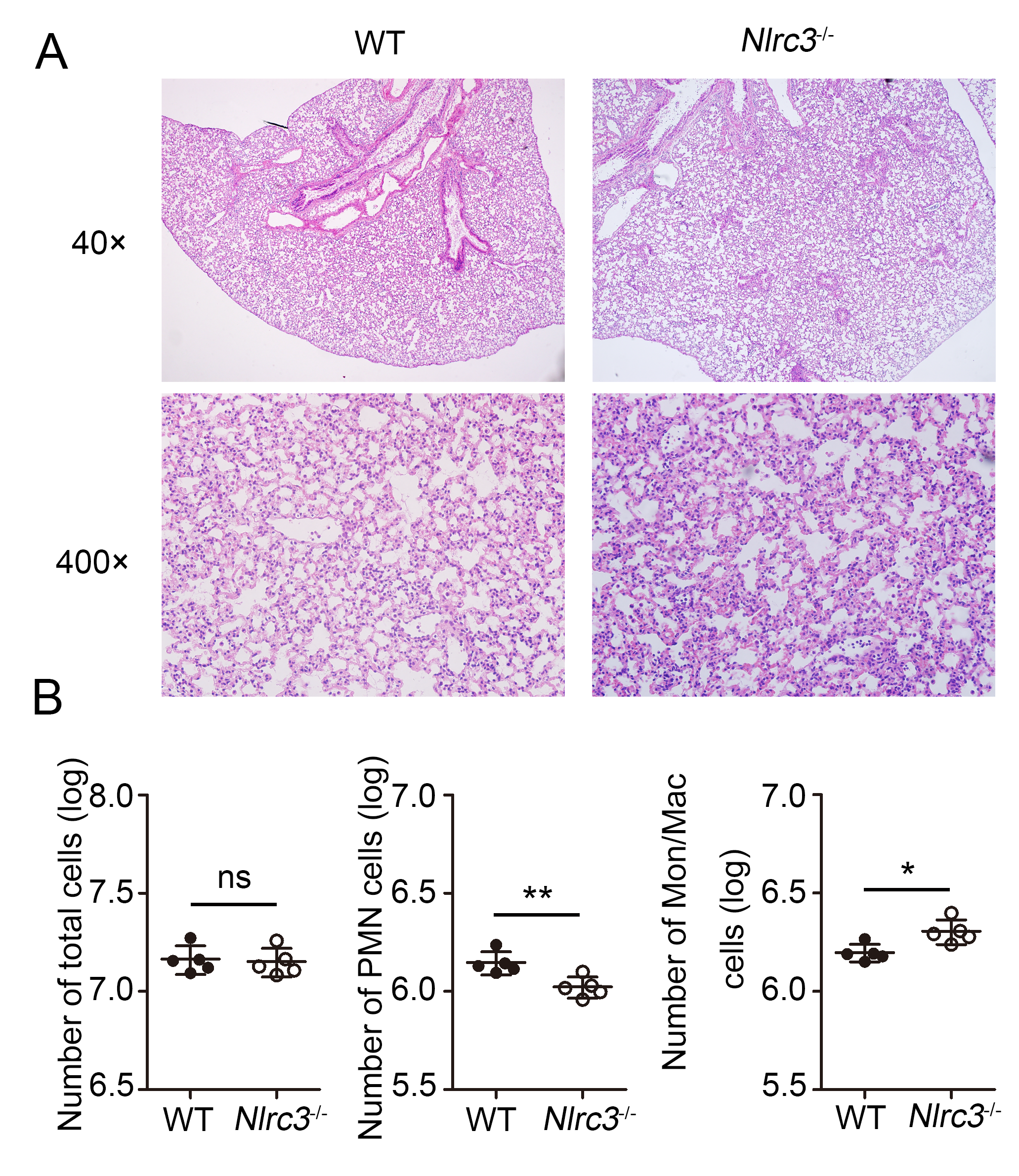

Supplement: S3 Fig — WT and Nlrc3-/- mice infected with approximately 200 colony-forming units of M. tuberculosis were monitored. (A) H&E-stained lung sections derived from two representative mice in each group of mice 3 w.p.i.. The magnification is shown at the right of each image. (B) Numbers of lung-infiltrating cells were counted at. Data shown are the mean ±SD. *P < 0.05 and **P < 0.01. Data are representative of three independent experiments with similar results. (TIF) [file ppat.1007266.s003.tif]

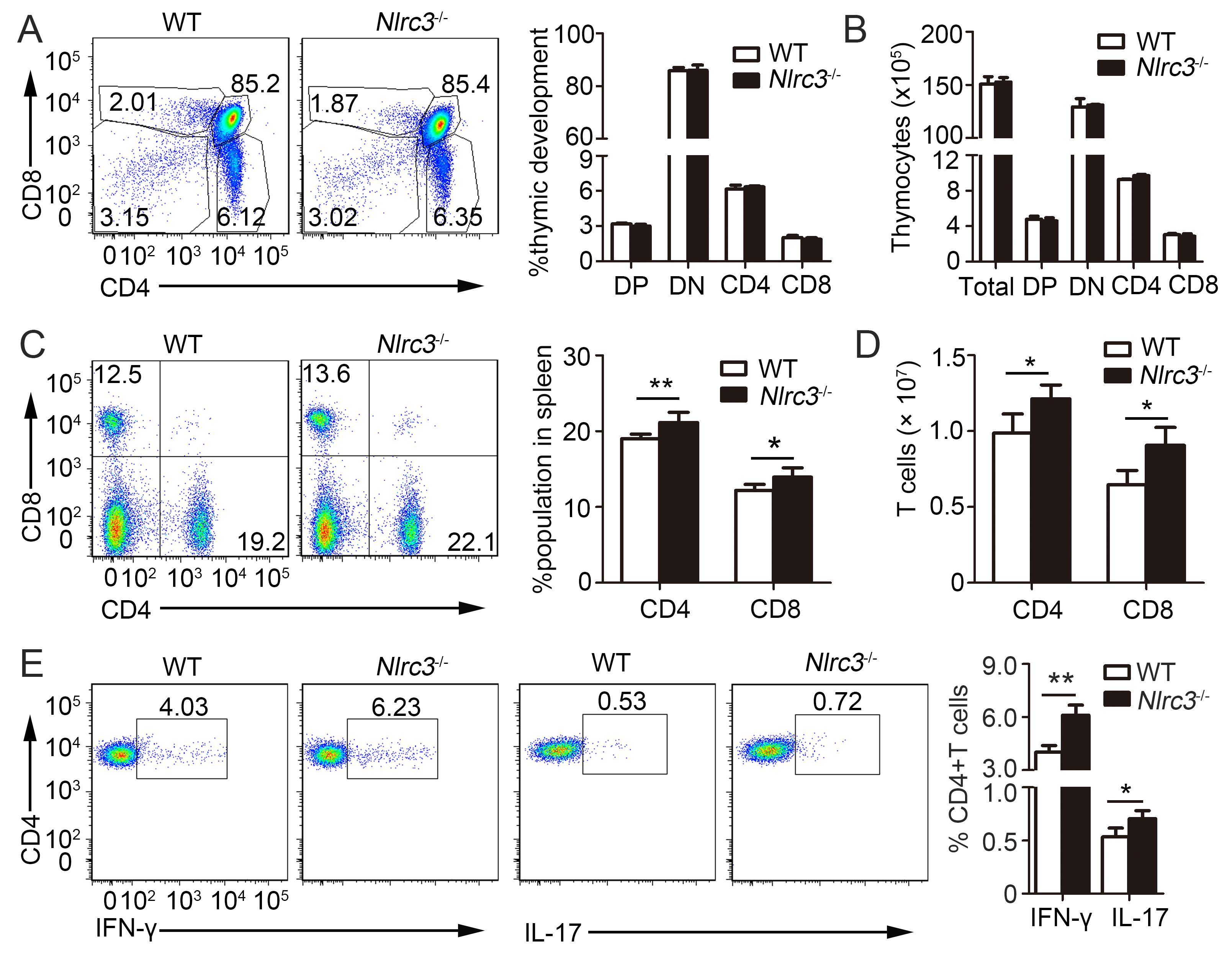

Supplement: S4 Fig — NLRC3 does not affect thymic development but does influence mature CD4+ T cells. (A) Representative expression of CD4 and CD8 by WT and Nlrc3-/- thymocytes. Pooled data are presented in the right panel. DN, double negative (CD4- CD8-); DP, double positive (CD4+ CD8+); CD4, CD4 single positive (CD4+ CD8-); CD8, CD8 single positive (CD4- CD8+). (B) Total numbers of thymocytes in each stage of thymic development. (C) Representative expression of splenic CD4+ and CD8+ T cells from WT and Nlrc3-/- mice. Pooled data are presented in the right panel. (D) Enumeration of splenic CD4+ and CD8+ T cells from WT and Nlrc3-/- mice. (E) Production of IFN-γ and IL-17 by CD4+ T cells following stimulation with PMA/ionomycin. Pooled data are presented in the right panel. Data shown are the mean ±SD. *P < 0.05 and **P < 0.01. Data are representative of three independent experiments with similar results. (TIF) [file ppat.1007266.s004.tif]

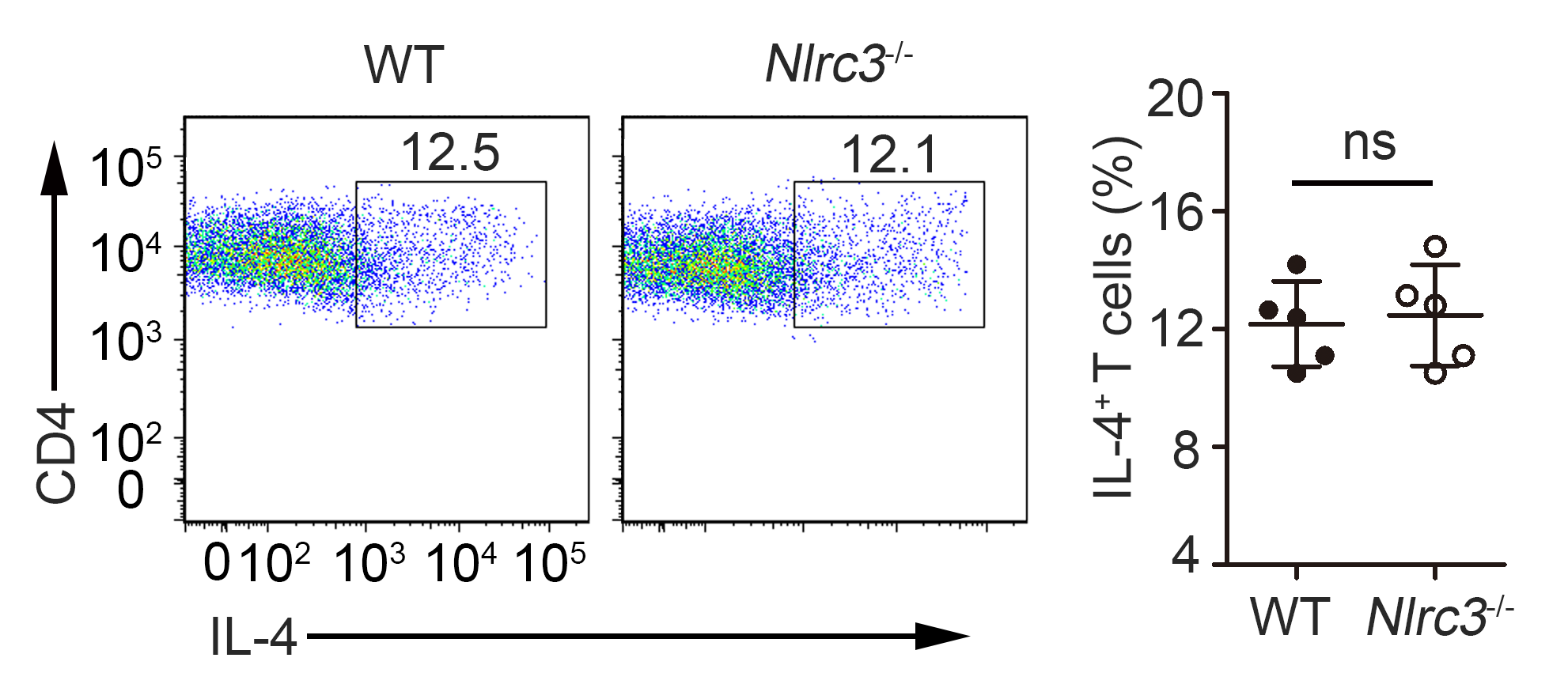

Supplement: S5 Fig — NLRC3 does not affect differentiation of Th2. Purified WT and Nlrc3-/- naïve CD4+ T cells were polarized in Th2 culture conditions for 4 days. Data shown are the mean ±SD. Data are representative of three independent experiments with similar results. (TIF) [file ppat.1007266.s005.tif]

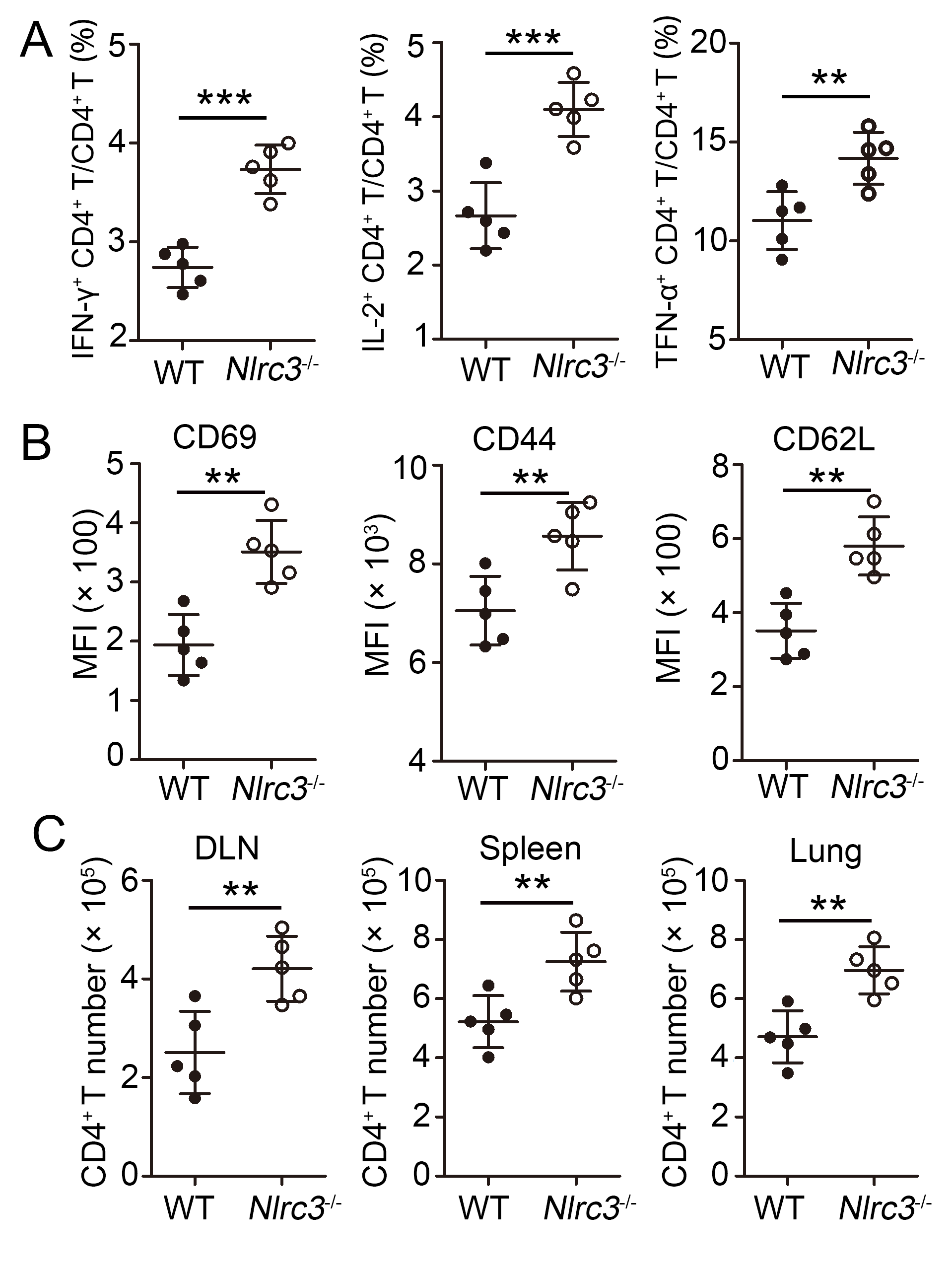

Supplement: S6 Fig — NLRC3 deficiency of CD4+ T promotes the antibacterial ability in vivo. Purified WT or Nlrc3-/- naïve CD4+ T cells were adoptively transferred into Rag2-/- mice. Then recipient mice were infected with M. tuberculosis and parts of mice were harvested at 3w.p.i.. (A) Lung cells were restimulated with M. tuberculosis lysate directly ex vivo and the intracellular production of IFN-γ, IL-2, and TNF-α by CD4+ T cells was determined. Pooled data are presented. (B) Mean fluorescence intensity (MFI) of activation markers by lung CD4+ T cells. (C) Enumeration of CD4+ cells in draining lymph nodes (DLNs), spleens and lungs. Data shown are the mean ±SD. **P < 0.01 and ***P < 0.001. Data are representative of three independent experiments with similar results. (TIF) [file ppat.1007266.s006.tif]

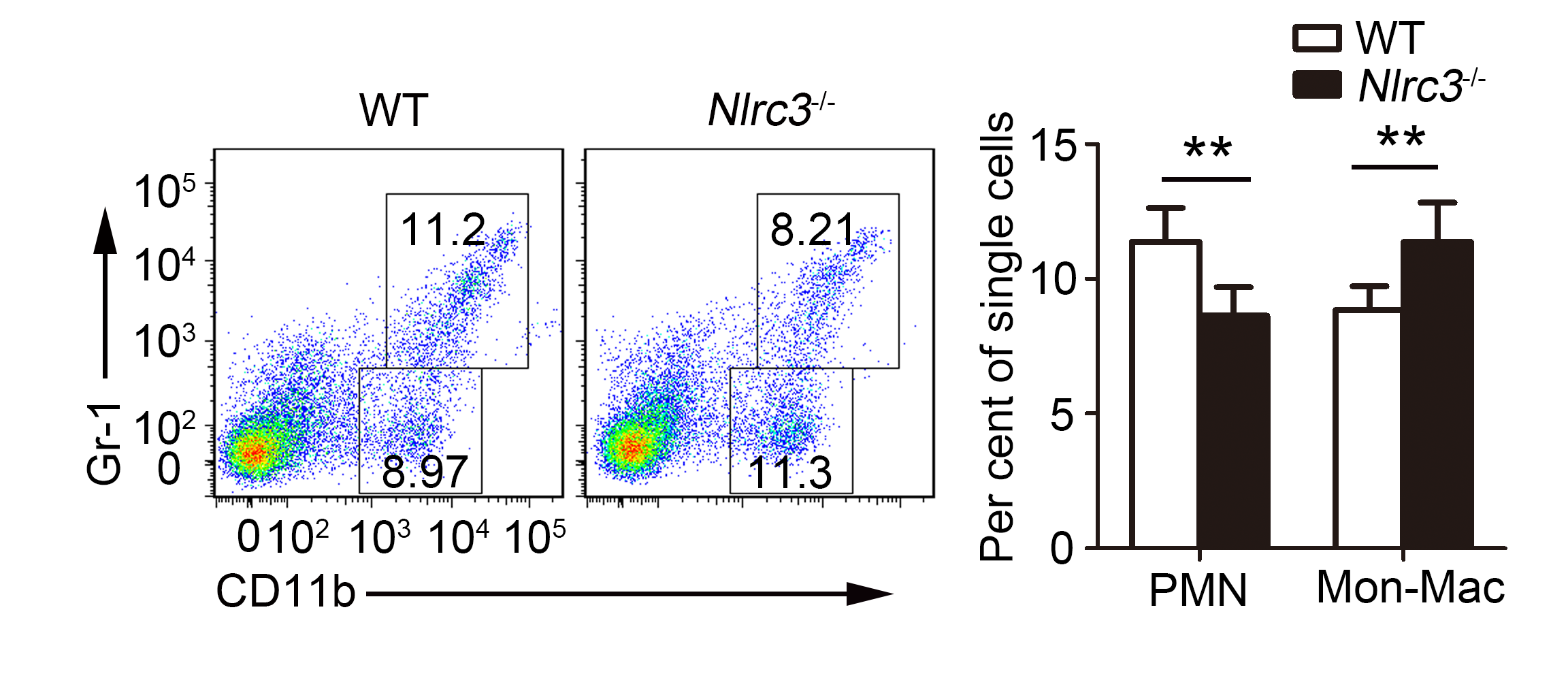

Supplement: S7 Fig — NLRC3 deficiency of CD4+ T affected infiltration of myeloid cells to lung. Purified WT or Nlrc3-/- naïve CD4+ T cells were adoptively transferred into Rag2-/- mice. Then recipient mice were infected with M. tuberculosis and parts of mice were harvested at 3w.p.i.. Frequencies of lung-infiltrating cells that are neutrophils (CD11b+ Gr-1+) or monocyte-macrophages (CD11b+ Gr-1-). Pooled data are presented in the right panel. Data shown are the mean ±SD. **P < 0.01. Data are representative of three independent experiments with similar results. (TIF) [file ppat.1007266.s007.tif]

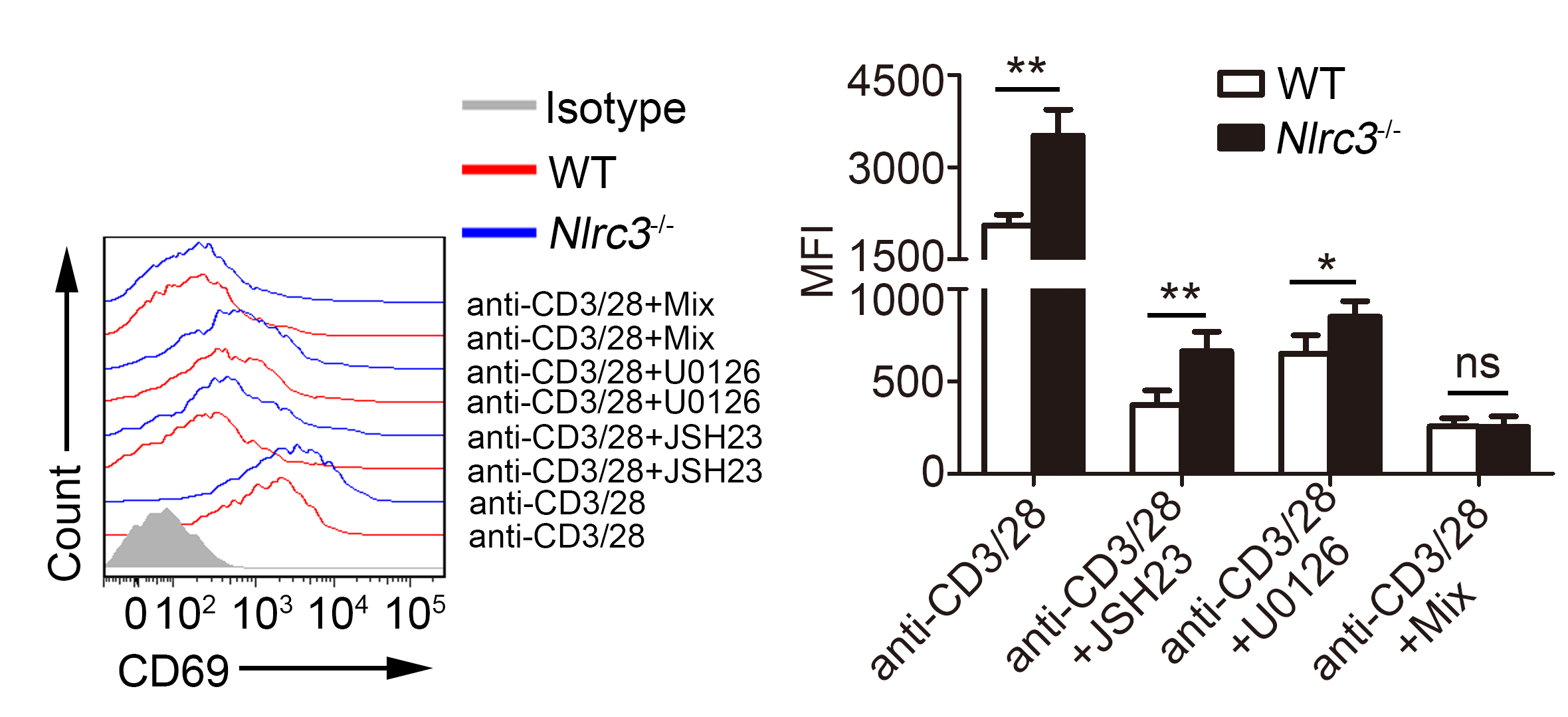

Supplement: S8 Fig — NLRC3 suppresses activation of CD4+ T cells via negatively regulating NF-κB and ERK Signaling. Purified WT and Nlrc3-/- CD4+ T cells were stimulated for 48 hr with anti-CD3 (1 μg/ml) plus anti-CD28 (1 μg/ml) in the presence or absence of the NF-κB inhibitor JSH-23 (20 μM), MEK1/2-inhibitor U0126 (40 μM) or the mix of the two. And then expression of CD69 were detected. *P < 0.05 and **P < 0.01. Data are representative of three independent experiments with similar results. (TIF) [file ppat.1007266.s008.tif]
